# Supplementary material for: Fish provision in a changing environment: The buffering effect of regional trade networks
Source: PLoS One. 2021 Dec 20;16(12):e0261514. doi: 10.1371/journal.pone.0261514 (PMC8687593; doi:10.1371/journal.pone.0261514)
Supplement: S1 Appendix — (DOCX) [file pone.0261514.s001.docx]

**Supplementary information 1: ODD+D protocol**

The Small-Trade Model

Here we present the model description according to the ODD (Overview, Design concepts, Details) protocol including human decision making (ODD + D) for describing agent-based models [1–3]. The Small-Trade model and this protocol build on the FishMob model [4], but go substantially beyond.

Link to the model and publication at CoMSES for review [5].

Table of Contents

[1. Purpose & Patterns 2](#_Toc75419689)

[2. Entities, state variables, and scales 3](#_Toc75419690)

[3. Process overview and scheduling 5](#_Toc75419691)

[Motivations 5](#_Toc75419692)

[4. Design concepts 5](#_Toc75419693)

[Basic principles: Theoretical and empirical background 5](#_Toc75419694)

[Adaptation & Individual decision-making 6](#_Toc75419695)

[Objectives 6](#_Toc75419696)

[Emergence 6](#_Toc75419697)

[Learning 6](#_Toc75419698)

[Prediction 6](#_Toc75419699)

[Sensing 7](#_Toc75419700)

[Interactions 7](#_Toc75419701)

[Stochasticity 7](#_Toc75419702)

[Collectives 7](#_Toc75419703)

[Heterogeneity 7](#_Toc75419704)

[Observation 8](#_Toc75419705)

[Implementation 9](#_Toc75419706)

[5. Initialization 9](#_Toc75419707)

[Model experiments: different Network structures 10](#_Toc75419708)

[Environmental Scenarios 10](#_Toc75419709)

[6. Input data 12](#_Toc75419710)

[7. Submodels 12](#_Toc75419711)

[SM1. Set vertical demand for traders 14](#_Toc75419712)

[SM2. Dealers predict what fish they need and request it 15](#_Toc75419713)

[SM3. Update fish demand 15](#_Toc75419714)

[SM4. Update species catchability 15](#_Toc75419715)

[SM5. Allocate fishing effort 16](#_Toc75419716)

[SM6. Fishing 18](#_Toc75419717)

[SM7. Update catch 19](#_Toc75419718)

[SM8. Update Stocks 19](#_Toc75419719)

[SM9. Dealers update actual request 20](#_Toc75419720)

[SM10. Traders sell to dealers (horiz. trade) 20](#_Toc75419721)

[SM11. Traders offer fish and dealers buy (horiz. trade cont.) 21](#_Toc75419722)

[SM12. Trading to final market 22](#_Toc75419723)

[References 22](#_Toc75419724)

# 1. Purpose & Patterns

The aim of the Small-Trade model is to understand how trade networks consisting on different types of local traders’ influence fish provision in a multi-market context; and identify potential mechanisms on how different outcomes emerge. We operationalize fish provision in the model by measuring outcomes at the macro level (i.e. fish scarcity in two different markets, waste, stock exploitation) and at the micro level (i.e. traders’ supply and supply variability). In particular, the model answers the following overarching question: *how do local trade networks influence the availability of fish in situations of high catch variability as found in spatially heterogenous, multi-species fisheries?*

The Small-Trade model is an empirically-informed model, based on the study case of Baja California Sur (BCS), Mexico. The types of traders, network structures and processes included in the model are empirically informed as explained in the main manuscript. However, some of the model components, such as the representation of different fish populations and the interactions between traders and fish populations are more stylized, aiming to represent a generalized empirical context of a spatially heterogenous, multi-species and multi-market small-scale fishery.

The model is designed for scientist and practitioners interested in better understanding the importance of trade networks in small-scale fisheries. It provides a virtual setting for theoretical and empirical exploration of the influence of different trader types, trade network structures, and catch and market dynamics, affecting two fishery regions that can be connected through trade, and yields specific considerations and hypothesis for future empirical research.

A pattern that can be found both in the literature and in the BCS study case, is the increased recognition of the importance of local trade networks in small-scale fisheries, where such networks have been described in multiple small-scale fisheries across the globe. This model contributes to a research gap investigating the influence of local trade networks, and thus pre- and post- harvesting activities and processes in small-scale fisheries, on the availability and provision of fish. In doing so, the model specifically accounts for the spatial component of regional trade networks, which are supplied by heterogenous fish production regions that can be subject to similar or different catch dynamics.

# 2. Entities, state variables, and scales

Fish populations in the model represent two different types of species (named LV (Low Value) and HV (High Value)) each of which is only sold to one type of market (LV and HV respectively). This corresponds to species types and markets A (LV) and B (HV) in the manuscript. In this model the types do not have different values, but they cannot be sold to the respective other market because they represent cases of non-substitutable species which often exist in fisheries. For example in Baja California Sur, red snapper and triggerfish are rarely substituted [17]. There are two fishing regions, which each have populations of both types.

There are two types of traders; dealers and sellers. Dealers can request and buy fish from other dealers/sellers and sell it, while sellers can only sell fish. Traders are connected through a trade network. We refer to this network and the associated trade processes as horizontal trade in the model. All traders can also sell fish to the markets through what we name “vertical trade”. Figure 1 one shows this model structure, including all types of agents in the model and their interactions.

There are two buyers implemented as agents, one that buys HV fish from traders and sells to the HV market, and one buys LV fish and sells to the LV market; and all traders can sell to both buyers. Buyers have a fixed demand for the species they are dealing and will not buy more than they demand. Buyers represent the market in this manuscript.

Every trader belongs to only one of the two regions. There are the same number of traders in each region and the same proportion of the two trader types per region (unless there is an uneven number of dealers and sellers). Regions and fish populations are ecologically independent.


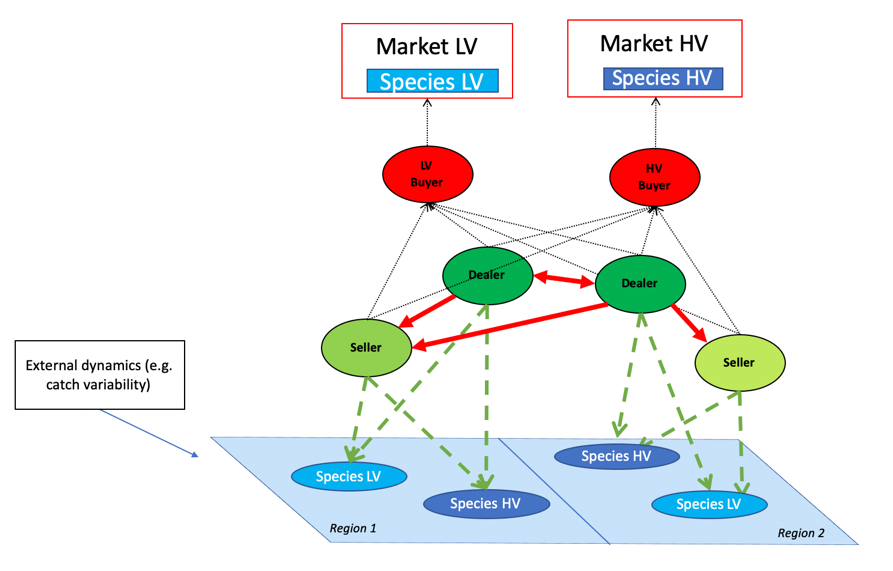


Figure 1. Model structure. The two types of traders are represented in green. They target two types of species, can trade with each other, and sell the catch to the buyers or markets. there are two fish stocks (populations) in each of the two regions, one of each type. Traders belong to only one of the two fishing regions each. red arrows indicate the trade network, where directionality (Dealer🡪Seller) indicates dealer “request to”, or “buy from”, Seller.

The main global variables are detailed in table 1. Other variables of interest for the model processes are detailed in the subsequent sections.

Table 1. Global variables in the model.

| Variable name | Variable type and unit | Meaning & why chosen |
| --- | --- | --- |
| steps | Discrete; number | Number of time steps in weeks. Fishing occurs during the week and is sold to traders, which is aggregated into one timestep. Trader’s transport of fish and buying/selling of fish does not usually take place daily, occurring with a varying frequency (e.g. 1-3 times per week). |
| years | Real; years | How many years have passed in the simulation. A default simulation runs over 20 years. Chosen based on that overfishing of a resource can take decades, and management can run over several years. The timeline also enables to the model dynamics to stabilize. |
| Market demand | Calibrated; Tons | Demand from the markets. Fishing and trading provide fish to satisfy different market demands, from local to global. In this version of the model there is a constant demand calibrated in relation to the fishing effort to exploit the fish stocks at Maximum Sustainable Yield, equal for the two market types. |

#

# 3. Process overview and scheduling

There are two different types of processes in the model, those related to fishing (blue, Figure 2), and those related to trading. Related to trading, the first steps indicate information transmission about demand and supply, where traders predict their need for fish (orange, Figure 2); and finally the actual trade where fish is bought and sold between the different actors and the market (yellow, Figure 2). Figure 2 indicates the flowchart following the description of the model process in the main manuscript. See submodels (section 7 below) for more details on each step.

***
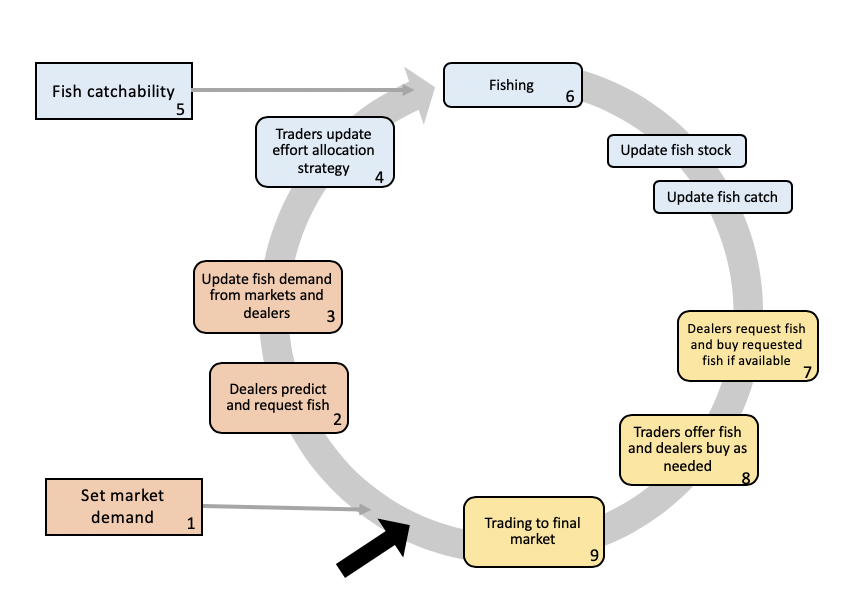
***

Figure 2. Flow chart of the model process. black arrow indicates where the process starts.

## Motivations

The qualitative analysis of semi-structured interviews conducted in 2019 informed the specification of the different model processes. This motivation is detailed in table 1 in the main manuscript [7] and the empirical findings are further described in supplementary material 2.

# 4. Design concepts

## Basic principles: Theoretical and empirical background

The overall principle underlying the model is based on understanding trade networks in small-scale fishing, by explicitly accounting for different trading actors and their social and social-ecological relationships. Therefore trade is conceptualized within a social-ecological system [8], and we specifically build on existing literature describing fisher-trader and trader-trader relationships in small-scale fisheries’ value chains from a social-ecological perspective [9–11]. The inclusion of a stable trade network is based on the understanding of trade as constituted by social relationships instead of an economic process based on price setting mechanism, following the theory of embeddedness within economic sociology [12].

**Fish stock dynamics** are modelled following classic fisheries bioeconomic modeling [13], particularly the Gordon-Schaeffer growth model.

The decision-making of the agents is based on assumptions that were derived from a qualitative analysis of semi-structured interviews with traders in Baja California Sur, Mexico [7] and decided on in relation to the research question asked in this model. Model processes and network structures were based on this data, as well as previous empirical information of the study case and broader literature of fisheries value chains in other study cases. Data was mainly available at the level of traders and regarding the trade structure, and no data was available regarding the model outcomes (i.e. food scarcity and waste at the markets, stock exploitation level).

## Adaptation & Individual decision-making

Traders are the agents that make decisions in the model. Traders aim to satisfy their demand for fish, including satisfying the requests of other traders that are linked to them since these links represent stable and committed trade relationships. In addition, they also aim to increase their Catch Per Unit Effort (CPUE) and not to allocate effort to species that are not “biting” or being caught. Thus, when deciding how to allocate their limited effort between the two species, they balance the aim to satisfy their demand with their knowledge of changes in their CPUE. Traders adapt their effort allocation based on their previous CPUE, previous demands from others, and current demand from the final market. Spatial aspects do not influence the decision-making process per se, but can indirectly influence the outcomes and decision-making since there can be different availability of fish in different regions, influencing the catch of each trader. Social norms or cultural values do not play an explicit role in decision-making besides the strong commitment between traders which is implicitly related to social norms.

## Objectives

The objective for the traders is to satisfy their individual market demand each time step, while also accounting for the CPUE. There is no long-term objective.

## Emergence

Agents interactions lead to emergent patterns of fish availability at the market level including fish scarcity or waste of each species. The Stock exploitation level also emerges as a result of individual decisions on how to allocate fishing effort.

## Learning

Individual or collective learning is not represented.

## Prediction

Traders predict how much fish they would need to decide how to allocate their effort. The prediction is based on their catch in the previous time step, and the demand (fish requested) by their traders also in the previous time step. Thus, their prediction can be somewhat erroneous, since their catch may change (as a consequence of changes in catchability or stock dynamics) and the received demand may change based on what each trader caught.

## Sensing

Traders and buyers sense the market demand without error. Traders know the predicted and actual requests of the other traders that are linked to them without error. The mechanisms through which traders get this information is modelled explicitly in the information transmission and actual trade strategies. There is no temporality in sensing, or explicit cost for obtaining information, but the network structure (e.g. number of links of each agent) will influence trader’s capacity to get information and trade with other traders. The network structure will also influence the “quality” of that information, because the amount of fish requested by each trader to each of his/her trading partners depends on his/her number of links. Thus traders do not receive information about how much fish the trading partners will need in total, unless they are the only ones having a link to them.

## Interactions

Traders interact with all other traders based on their directed trade network every time step. The trade network is static and imposed by the researcher. The network determines the possibilities for the exchange of information regarding the demand of each fish species, and the traded fish, further detailed in the submodels section (SM2). Dealers predict what fish they need and request it, SM10. Traders sell to dealers). In addition, all traders interact with markets (as they sell to markets, see “SM12. Trading to final market”) and with two types of fish species (go fishing submodel “SM6. Fishing”).

## Stochasticity

Traders are randomly distributed to two regions, with eight traders in each region as well as equal number of trader types per region. The generation of theoretical random networks in the sensitivity analysis also involves randomization based on a given probability of creating links.

In the catch variability scenario, the catch varies for each trader each time step. The catchability for each trader (i.e. individual catchability) is multiplied by a value set each time step to the normal distribution around 1 and the standard deviation of 0.2.

## Collectives

Traders interact with each other in stable networks set up by the researcher at initialization. This could be viewed as a collective, but there are no other collectives in the model.

## Heterogeneity

Traders are heterogeneous through what area they belong to and if they are dealers or sellers (where dealers can request fish to others and buy fish from others, whereas the sellers can only sell fish). Traders also have different number of links to other traders. The Fish populations are heterogenous in their market type, type A fish are sold to market A, type B fish are sold to market B. Fish populations as well as the fishing regions can also be heterogenous when affected by different catchability dynamics and seasonality.

## Observation

Global, individual and group parameters are presented in plots in Netlogo and also saved to a cvs file every time step and analyzed in R [14], see Table 2.

Table 2. Data collected in each time step from the ABM for testing, understanding, and analyzing the model.

| Level | Variable | Description |
| --- | --- | --- |
| Global | ticks | Number of time steps passed |
| Global | total links | Number of links in the trade network |
| Global | Links traders 0 | Number of request links (out-links) going from region 1 to region 2 |
| Global | Links traders 1 | Number of request links (out-links) going from region 2 to region 1 |
| Global | homelinks0_out | Number of request links (out-links) within region 1 |
| Global | homelinks1_out | Number of request links (out-links) within region 2 |
| Regional | Market availability* | Scarcity of fish for each species, set to 0 when supply > demand |
| Regional | Waste* | Fish that is wasted (not sold) of each species, set to 0 when supply < demand |
| Regional | Exploitation level* | Index representing the mean exploitation level of each fish stock per region |
| Regional | Stock size | The total weight of the SSB per region |
| Individual (links) | Predicted request | What dealers predict they need from others in the information transmission process (dealers let their network know what they aim to buy from them) |
| Individual (links) | Final request | What dealers need from others (and hope buy if they have fish available) in the actual trade process |
| Individual (traders) | Dealer demand | What the dealers demand from other traders. |
| Individual (traders) | Fished supply | Fish supply (catch) traders got from fishing |
| Individual (traders) | Bought supply | Supply dealers bought from sellers or other dealers |
| Individual (traders) | Wasted supply | What traders cannot sell to the markets and goes to waste |
| Individual (traders) | Traders final supply* | What traders supply (sell) to dealers and markets *(Fished supply + Bought supply - Wasted supply)* |

**Value used as model outcome in the model analysis*

## Implementation

The model was implemented in Netlogo [15] version 6.1.2-beta2 , analyzed using R [14] and is accessible at CoMSES [5]

# 5. Initialization

The model can be initialized with different options found in the Netlogo interface. Here we describe the parameters used for the analysis conducted in this manuscript, even if further possibilities exist for model analysis and exploration.

The number of traders is set to 16 to match the empirically-informed networks but with an even number of actors, and can be selected in the variable *initial_n_traders.* Traders are distributed between the regions (*initial_distr_traders*), as “*even trader types*”, so that there is an equal number of traders per region and this maintains the proportion of dealers and sellers in each region. There are 2 markets ^[[1]](#footnote-1)^which represent each a market A and a market B. Markets are implemented as agents to expand on the vertical connectivity of the network in future model extensions.

There are 2 types of species per region (*num_local_species_per_subregion* = 2). The carrying capacity is the same for both species (*carryng_cap*=1000 1000) and there is no noise or variability in the stock levels (*stock_noise_var*=0).

The market demand can also be modified with different dynamics that can affect all or one of the species. Here we use a constant demand (*dmd_type*=eq) for both species (*dmd_mc*=ALL).

Additional options are presented in the submodels sections below.

## Model experiments: different Network structures

See supplementary material 2 in [7] for details on the generation of empirically-informed network structures. These structures are saved in “.graphml” file extensions using R [14] and imported using the “Nw” Netlogo extension (*http://ccl.northwestern.edu/netlogo/5.0/docs/nw.html*).

In addition to empirically informed network structures, three theoretical structures are used for comparison (Table 3).

Table 3. Variables and parameters defined in the different model experiments.^[[2]](#footnote-2)^

| Experiment type | Network type | Share sellers | Density | Meaning |
| --- | --- | --- | --- | --- |
| Theoretical | none | NA | NA | “absent” network where traders have no links |
| Theoretical | random | 0 | 1 | “High-connectivity” network, generated following an Erdos-Renyi random network where all traders are dealers |
| Theoretical | eq | 0 | NA | “pairs-across” networks where traders are linked pair-wise between regions where all traders are dealers |
| Empirically-informed | Simulated1 | NA | NA | “Empirically-simulated” network is imported |
| Empirically-informed | Simulated3 | NA | NA | “More-dealers” network is imported |

## Environmental Scenarios

Table 4 describes the variables and parameters that define each of the scenarios of the model at initialization. For each of these scenarios, the previous 5 experiments are simulated.

Table 4. Variables and parameters SETTINGS AND definITIONS in the two different model scenarios

| Variable name | Parameter | Meaning of variable (and scenario setting) | Motivation |
| --- | --- | --- | --- |
| Seasonality scenario | | |  |
| cb_type | Seasonal | Cb_type affects the seasonal species catchability. (seasonal species catchability = 0 for 6 months of the year, and seasonal species catchability = 1 during the other months) | We model two hypothetical seasons of the same duration, where the species is either catchable or not. Future model extensions can test the effect of cyclic patterns (where there are more progressive changes in catchability) with different periodicity. |
| regional_cb | R1 | Region that is affected by the cb_type (R1 = region 1 in the manuscript) | Seasonality is only affecting one region. Regions can be affected differently by environmental variability or institutions |
| species_cb | LV | Species that are affected by cb_type (LV= species A in manuscript) | Seasonality is only affecting LV species. Not all species are affected equally by environmental variability or institutions |
| catch_noise_  variability | 0 | Catch variability (there is no random variability in the catch received by traders) | There is no randomness in the catch received to better understand the effect of other parameters. |
| Catch variability scenario | | |  |
| cb_type | equilibrium | Cb_type affects the seasonal species catchability. (seasonal species catchability = 1) | Catchability can depend on external factors not affected by the region or species (e.g. weather) |
| regional_cb | ALL | Regions affected by cb_type (all) | All regions are affected equally by catchability |
| species_cb | ALL | Species affected by cb_type (all) | All species are affected equally by catchability |
| catch_noise_  variability | 0.2 | Catch variability set by trader´s individual catchability (there is 20% stochastic variability between the catch that is expected and the actual catch that is received) | There can be divergences between the amount of fish that traders expect or wish to get and the catch that is finally received, which is affected by fisher´s decisions and biological and ecological dynamics affecting fish populations, that are not always possible to predict. |

# 6. Input data

The quantitative input data are the network structures imported at initialization and described in section 5 above. See supplementary materials 1 and 2 in Gonzalez-Mon et al. [7] for details on the empirical data informed in the model.

# 7. Submodels

The following submodels are implemented in the model. These are the same as the processes depicted in the flow chart (figure 2). Table 5 shows the steps in the flow chart associated to the model procedures and the associated variables that are either used in or set by the procedures. The index *a* below denotes “actual” supply or request (for the actual trade), the index *p* denotes predicted (for the information transmission steps), the index *f* denotes fished supply, the index *b* denotes bought supply, the index *w* denotes waste, and t denotes timestep. The index *h* denotes horizontal trade and the index *v* vertical trade, where vertical trade is referred to as the trade with market, and horizontal as trade between dealers and sellers.

Table 5. Submodels, corresponding procedure on Netlogo, and related agent variables and links. SP species and BRS buyers. The last column specifies which variables are set by a procerure (Notated as “SETS”) and which variables are used by a procedure (notated as “USES”).

| Step/ submodel (SM) | Procedure name in Netlogo | Agent variables and links that are used or set by the procedure. |
| --- | --- | --- |
| SM1. Set market demand | traders-update-vertical-demand | SETS TRADER   - dmdTb ”LVv”   USES   - buyerdemandTb |
| SM2. Dealers predict what fish they need and request it | dealers-update-request ”p” | USES TRADER   - (fishedcatchTb)    (t-1)* - dmdTb ”LVv” (t) - dmdTb ”LVh” (t-1) - SupplyTb ”LVf”    (t-1)   SETS TRADER LINK   - requestLVp (on link)   *for avoiding to request fish from traders in own region if there is no fish there if request_scenario = “other_region” is TRUE |
| SM3. Update fish demand | (traders-update-horizontal-demand )  if network = TRUE | USES TRADER LINK   - [requestLVp] of my-in-traderlinks   SETS TRADER LINK   - DmdTb ”LVh” |
| SM4. Allocate fishing effort | traders-decide-effort | USES TRADER, SP   - fishedcatchTb the_spID - individualeffortTb the_spID - individualeffortEQTb the_spID - stock * speciescatchability - dmdTb ”LVv” - dmdTb ”LVh”   SETS TRADER   - individualeffortTb the_spID |
| SM5. Fish catchability | Species-update-catchability | SETS SP   - species-update-catchability |
| SM6. Fishing | traders-go-fish | SETS TRADER, SP   - fishedcatchTb   USES   - individualeffortTb   (resets fishedCatch and then sets it again) |
| SM7. Update catch | traders-update-fished-supply | USES TRADER   - fishedcatchTb   SETS TRADER   - supplyTb "LVf" - supplyTb "LVa" |
| SM8. Update stocks | update-stocks | USES TRADER   - fishedcatchTb   SETS SP   - stock |
| SM9. Dealers update actual request | dealers-update-request ”a” | ; a = actual |
| SM10. Dealers request fish and buy fish | traders-trade-with-dealers "S"   traders-trade-with-dealers "D" | USES TRADER LINK   - requestLVa (of buying trader to me) - Capital (me and buying trader)   SETS TRADER   - SupplyTb “LVa” (of buying trader) - SupplyTb "LVa" (of me)   SETS TRADER LINK   - requestHVr of (of buying trader to me) |
| SM11. Traders offer fish and dealers buy | dealers-continue-trade | USES TRADERS LINKS   - requestLVr   SETS   - SupplyTb “LVa” (of buying trader) - SupplyTb ”Lva” (of me) - Capital (me and buying trader) |
| SM12. Trading to final market | traders-trade-with-market | USES TRADERS, BRS   - supplyTb “LVa” - priceTb (of buyer) - buyerdemandTb (to check dmd full) - fleetcatchMCTb (to check dmd full)   - If flcatch > bdmd : not buying   SETS   - fleetcatchMCTb - supplyTbLVw    (waste) - incomeList - accumulatedIncome - capital |

## SM1. Set vertical demand for traders

In this submodel each trader receives the current demand from the markets, that the traders then will try to meet by fishing and trading with each other. The demand is however constant in the scenarios presented in Gonzalez-Mon et al. [7].

*traders-update-vertical-demand*

sum_boats = count boats of all traders

share_LV_per_boat = demand_LV / sum_boats

share_HV_per_boat = demand_HV / sum_boatsfor each trader [

dmdTb "LVv" = my_num_boats * share_LV_per_boat

dmdTb "HVv" = my_num_boats * share_HV_per_boat

]

## SM2. Dealers predict what fish they need and request it

In this model we use a request model setting *request_scenario* to ALL. This means that traders distribute their request equally amongst all their trade partners, even if they do not have fish. This takes into account the number of boats per trader, but is set to equal for all traders in the model. *request_scenario = other region* is a possibility that allows traders to request fish only to traders from the other region that are linked to them, and therefore target the request more efficiently when there is lack of fish in one region. This option is included for model testing and understanding and can be further developed into more adaptive request strategies.

The pseudo code is for *request_scenario* to ALL. *request_scenario = other* region it works the same but the request for a species is only done to traders in the other region if the catch is zero in a dealer’s home region.

*dealers-update-request (request_type)*

traders_requesting = dealers with links to other traders

FOR ALL TRADERS_requesting [

LV_catch_my_region = catch of LV in my home region

HV_catch_my_region = catch of HV in my home region

My_request_LV = vertical request this timestep LV +

horizontal request previous time step LV – fished supply previous timestep LV

My_request_HV = vertical request this timestep HV +

horizontal request previous time step HV – fished supply previous timestep HV

num_trader_boats_LV = sum all boats of my trader links

num_trader_boats_HV = sum all boats of my trader links

FOR ALL traders-to-request-LV-from [

request_LV = request_per_boat_LV * num_boats_other_trader

requestLVp = max (0, request_LV)

]

FOR ALL traders-to-request-HV-from [

request_HV = request_per_boat_HV * num_boats_other_trader

requestHVp = max (0, request_HV)

]

]

## SM3. Update fish demand

This submodel updates the total request from all my dealers by summarizing the predicted request of all my links to a dealer.

*Traders-update-horizontal-demand*

FOR ALL traders [

DmdTb "LVh" = sum [requestLVp] of my-in-traderlinks

DmdTb "HVh" = sum [requestHVp] of my-in-traderlinks

]

## SM4. Update species catchability

*Species-update-catchability*

Depending on which type of species catchability is chosen in the interface the catchability of the species is updated accordingly. In the associated publication [7] seasonal catchability is used in the seasonality scenario.

Seasonal species catchability (sc):

Every 6 months the species changes from catchable to non-catchable.

## SM5. Allocate fishing effort

The decision-making function first calculates the optimal effort to meet the demands of species A and B respectively, as well as that the optimal effort for maximizing the catch per unit effort for species A and species B (therefore balancing effort between the two decision-making models). Then the agent tries to optimize its effort per species A and B respectively to minimize the difference in demand and its catch for each species, as well as maximizing its catch per unit effort per species. This is done as follows.

$$\mathrm{If}\mathrm{de}_{A}>\mathrm{pe}_{A} \mathrm{then}e_{A}=\mathrm{de}_{A}-\mathrm{mag}_{A}$$

$$\mathrm{If}\mathrm{de}_{A}<\mathrm{pe}_{A}\mathrm{then} e_{A}=\mathrm{de}_{A}+\mathrm{mag}_{A}$$

$$\mathrm{If}\mathrm{de}_{A}=\mathrm{pe}_{A}\mathrm{then}e_{A}=\mathrm{de}_{A}$$

where

$\mathrm{mag}_{A}=br\times abs \left( \mathrm{de}_{A}-\mathrm{pe}_{A} \right)$

Where de_A_ denotes the optimal effort based on meeting the demand for species A according to the demand-driven sub-model; pe_A_, the optimal effort based on maximizing the CPUE for species A according to the CPUE-driven sub-model. The influence, or weight, of each sub-model is determined by a balance rate (br, with range from 0 to 1). mag_A_ represents the magnitude of the effort difference between the demand versus the CPUE optimal efforts. e_A_ denotes the new balanced effort for species A. This decision-making algorithm is repeated for species B, where e_B_ denotes the new balanced effort for species B. The final effort is then balanced between the species in relation to E:

$e_{A\_new}=\frac{E}{\left( e_{A}+e_{B} \right)} e_{A}$

$$e_{B\_new}=\frac{E}{(e_{A}+e_{B})} e_{B}$$

where e_A_new_ and e_B_new_ are the actual efforts applied for species A and B respectively, and E is the maximum effort that the trader can apply and need to share between species A and B.

The balancing effort between two decision-making models (or strategies), can de described in more detail in 4 steps as follows:

1. EFFORT PREVIOUS ROUND

Stored in variables: *tot_eff_LV, tot_eff_HV*

1. CALCULATE STRATEGY 1 (i.e. cpue-driven effort)

Calculate catch per unit effort:

my_cpue_LV = my_catch_LV / my_effort_LV
my_cpue_HV = my_catch_HV / my_effort_HV

Normalize the cpue difference between LV and HV:

Sum_cpue = my_cpue_LV + my_cpue_HV
the_norm_diff = ( abs (my_cpue_HV - my_cpue_LV) ) / Sum_cpue

Calculate at what rate to increase the effort for the LV species and then reduce for the HV species accordingly:

rate_of_change = min (mc_switch_rate, the_norm_diff)

The market class switch rate (mc_switch_rate) is a parameter that determines the maximum value of how much to switch your effort between species in one time step.

IF (my_cpue_LV > my_cpue_HV) [

the_inc = max (0, ( ( OLD_eff_HV * ( 1 + rate_of_change ) ) - OLD_eff_HV )

IF (the_inc = 0) [
 the_inc = ( OLD_eff_LV * ( 1 + rate_of_change) ) - OLD_eff_LV
 ]

tmp_eff_HV = OLD_eff_HV - the_inc
 tmp_eff_LV = OLD_eff_LV + the_inc

]

The temporary variables *tmp_eff_LV, tmp_eff_HV* now contains the new effort based on CPUE with an incremental change towards the species generating the most CPUE.

1. CALCULATE STRATEGY 2 (i.e. dmd-driven effort)

Sum the horizontal and vertical demand:

my_dmd_LV = dmdTb “LVv” + dmdTb “LVh”
my_dmd_HV = dmdTb “HVv” + dmdTb “HVh”

Calculate difference between this demand and catch obtained in previous round:

my_dmd_value_LV = my_dmd_LV – tot_catch_LV        
my_dmd_value_HV = my_dmd_HV – tot_catch_HV

Decide effort:

my_dmd_value_LV and my_dmd_value_HV are compared and a decision on new effort is taken and stored in variables:

dmd_eff_LV, dmd_eff_HV (new effort based on incremental change)

1. DECIDE FINAL STRATEGY BASED ON STRATEGY 1 AND STRATEGY 2

First calculate the differences between the low value efforts that the above calculations have given us:

part = balance_rate * abs (dmd_eff_LV – tmp_eff_LV) ;; eg 0.5 * 0.00002
base_eL = dmd_eff_LV ;; our dmd eff is the base that we work from
base_eH = dmd_eff_HV
new_eL = base_eL               ;; new effort is initiated to now the dmd eff
new_eH = base_eH

Set low value effort:

IF (dmd_eff_LV > tmp_eff_LV) [  ;; if the dmd eff is larger than the cpue effort
  new_eL = base_eL – part     ;; we want to reduce the dmd effort

]

IF (dmd_eff_LV < tmp_eff_LV) [ ;; if the dmd eff is lsmaller than the cpue effort
 new_eL = base_eL + part  ;; we want to increase the dmd effort

]

;; Set high value effort (new_eH) in the same way as above.

Calculate total effort:

EtotEQ = the total calibrated effort at model setup.

Because the new total effort should be the same as the calibrated effort (fishers are assumed not changing effort but instead choosing what species to fish more of) we weight up or down the new effort to sum up to the calibrated effort.

X = EtotEQ / (new_eL + new_eH)

new_eff_LV = x * new_eL

new_eff_HV = x * new_eH

## SM6. Fishing

Traders’ catch is calculated as:

*catch_tr_s_t_ = sc_t_ x ic_t_ x effort_tr_s_t_ x stock_s_t_*

Where the indices *tr* represents a trader, *s* represents the species and *t* the time step. For *sc* and *ic* see Table 6. All fishers are assumed to go fish every time step. The model is calibrated so that the fishing effort per region maintains sustainable fish resources. Only when traders start to request fish to others and/or decide to fish more of LV than of HV or vice versa, over exploitation can occur.

Table 6. Parameters for calculating catch.

| Parameter | Value | Description | Explanation |
| --- | --- | --- | --- |
| sc | 0 or 1 | Seasonal species catchability: If the species is catchable or not in a certain month | Some species appear (or are only biting) seasonally. This is a Boolean parameter set for each month. |
| ic | 1 with SD 0 or 0.2 | Individual catchability: A trader variable that changes each week to influence a trader’s individual catch | Individual catchability is set weekly, being normally distributed with mean = 1 and SD = 0.2 in the catchability scenario, to represent random individual variability in fish catches. |

## SM7. Update catch

Catch is used as trader’s supply (together with bought fish).

Supply_f_LV = fished LV catch

Supply_f_HV = fished HV catch

## SM8. Update Stocks

We represent the growth of each species in the model as a logistic growth minus the harvests extracted by all the traders (*totalcatch*) (Gordon-Schaeffer model). The key parameters are carrying capacity and growth rate of the fish species. The new stock of a species is calculated as:

*s_t+1_ = s_t_ + r s_t_ (1 – s_t_ / K) – totalcatch_t_*

*K* and *r* are set according to landings data of finfish in the Gulf of California that is then generalized to represent an archetypical stock of finfish (according to the explanation in Table 7, row 1 and 2). Table 7 gives an overview of the parameter values, descriptions and motivations for choosing them.

Table 7. Model parameters, their initial values, description and motivation or source for values chosen.

| Parameter | Value | Description | Explanation |
| --- | --- | --- | --- |
| r_1…4_ | {0.42, 0.42, 0.42, 0.42} | Growth rate of the fish populations | Based on the median of finfish of the 11 landing sites in the Gulf of California [6]. The empirical data is actual *r* per species (following the FishMob model [16] ) |
| K_1…4_ | {1000, 1000, 1000, 1000} | Carrying capacity (in tons) of fish stocks in each region for each LV and HV. | Stylized values set within the range in tons on of finfish landing sites in the Gulf of California based on values from [6]. We set all values to the same to avoid them influencing the results of our simulations, but still in the empirical range of tonnes landed. However, the way the model is calibrated the actual value of K does not affect the model outcomes. |
| SSB_1…4_ | K/2 | Initial standing stock biomass (SSB) | At initialization we want the SSB to be at sustainable levels. The amount of standing stock biomass that generated the maximum sustainable yield is calculated as K/2. |
| E | r/2 | Total fishing effort | Set so that fishing will be sustainable over an infinite time horizon, i.e., keep the stock at the maximum sustainable yield. |
| e | E/Nr | Fishing effort per trader | Fishing effort is how much fish they try to catch (hours spend at sea) and is divided among the two species according to the decision-making algorithm. |

## SM9. Dealers update actual request

*dealers-update-request “a” (the a represents the actual request)*

The actual (of final request) as opposed to the predicted request is calculated. This is done in the same way as the predicted request but based on actual catch this time step. See submodel SM2.

## SM10. Traders sell to dealers (horiz. trade)

*Traders-trade-horizontally*

traders_selling = traders with links to dealers

;; all selling traders

FOR ALL traders_selling [

LVsupp = supplyTb “LVa”

HVsupp = supplyTb “HVa”

;; those buying from me (showing only for LV)

FOR ALL in-traderlink-neighbors

the_trader_needs_LV = requestLVa

IF (LVsupp > 0 AND the_trader_needs_LV > 0 [

let amountLV 0

IF (the_trader_needs_LV >= LVsupp [

set amountLV LVsupp

]

ELSE [

amountLV = the_trader_needs_LV

]

let cost amountLV * dealer_LVprice

;; Add to buyers supple

SupplyTb "LVa" = SupplyTb "LVa" + amountLV

;; keep track of the bought supple

SupplyTb "LVb" = SupplyTb "LVb" + amountLV

;; the buying dealer pays for the catch

capital = capital - cost

;; remove from seller

LVsupp = LVsupp - amountLV ;; the sellers original supply is discounted

LVsold = LVsold + amountLV ;; what the seller sold

;; update the sellers capital

capital = capital + cost ;; the sellers increase in capital

tot_income_seller = tot_income_seller + cost ;; the sellers increase in income

;; update the request for the selling trader

requestLVr = max (0, (the_trader_needs_LV - amountLV))

]

accumulatedIncome = accumulatedIncome + tot_income_seller

revenueHorizSoldFish = revenueHorizSoldFish + tot_income_seller

;; after the selling remove supply accordingly

supplyTb "LVa" = LVsupp ;; update supply with what I have left

prev_sold = supplyTb "LVs"

supplyTb "LVs" = LVsold + prev_sold ;; update sold supply

]

]

## SM11. Traders offer fish and dealers buy (horiz. trade cont.)

*traders-trade-horizontally-continued*

This submodel is only used when *trade_scenario = continuous*. Showing for LV only to make it easier to follow, and as for HV the same things takes place.

traders_selling = traders with links to dealers

FOR ALL (traders_selling) [

LVsupp = supplyTb “Lva”

Lvsold = 0

tot_income_seller = 0

my_dealers = the dealers I am connected to

FOR ALL my_dealers [

Lvneeded = my_dealers remaining request

IF (Lvsupp > 0 AND Lvneeded > 0) [

IF (Lvneeded >= Lvsupp) [

amountLV = Lvsupp

]

ELSE [

amountLV = Lvneeded

]

cost = amountLV * dealer_Lvprice

;;FOR BUYER

SupplyTb “Lva” = SupplyTb “Lva” + amountLV

SupplyTb “LVb” = SupplyTb “LVb” + amountLV

capital = capital – cost

costHorizBoughtFish = costHorizBoughtFish + cost

;; FOR SELLER

Lvsupp = Lvsupp – amountLV

Lvsold = Lvsold + amountLV

capital = capital + cost

tot_income_seller = tot_income_seller + cost

]

]

;; (Repeat the pseudocode above for following the HV species trade)

;; After all trading is done, update additional variables

accumulatedIncome = accumulatedIncome + tot_income_seller

revenueHorizSoldFish = revenueHorizSoldFish + tot_income_seller

supplyTb "LVa" = LVsupp

supplyTb "LVs" = LVsold + supplyTb "LVs"

]

## SM12. Trading to final market

*traders-trade-vertically*

This submodel accounts for the limited capacity to buy fish in the final market, and thus here waste is calculates ( *Lim_trade_dmd = ON enables this feature in the NetLogo interface).* The supply that is higher than the final markets’ demand goes to waste instead of being sold. *Lim_trade_dmd = OFF* allows modelling a trade process where traders are always able to sell all of their catch irrespective of the demand level.

# References

1. Grimm V, Berger U, Bastiansen F, Eliassen S, Ginot V, Giske J, et al. A standard protocol for describing individual-based and agent-based models. Ecol Model. 2006;198: 115–126. doi:10.1016/j.ecolmodel.2006.04.023

2. Müller B, Bohn F, Dreßler G, Groeneveld J, Klassert C, Martin R, et al. Describing human decisions in agent-based models - ODD+D, an extension of the ODD protocol. Environ Model Softw. 2013;48: 37–48. doi:10.1016/j.envsoft.2013.06.003

3. Grimm V, Railsback SF, Vincenot CE, Berger U, Gallagher C, Deangelis DL, et al. The ODD protocol for describing agent-based and other simulation models: A second update to improve clarity, replication, and structural realism. Jasss. 2020;23. doi:10.18564/jasss.4259

4. Lindkvist E. FishMob: Interactions between fisher mobility and spatial resource heterogeneity (Version 1.0.0). CoMSES Comput Model Libr. 2020.

5. Lindkvist E. and González-Mon. Small-Trade Model: Exploring the role of trade networks in small-scale fisheries for regional fish provision. In: CoMSES Computer Model Library. 2021. doi.org/10.25937/n9z9-8n29

6. Giron-Nava A, Johnson AF, Cisneros-Montemayor AM, Aburto-Oropeza O. Managing at Maximum Sustainable Yield does not ensure economic well-being for artisanal fishers. Fish Fish. 2018; 1–10. doi:10.1111/faf.12332

7. González-Mon B, Lindkvist E, Bodin Ö, Zepeda-Domínguez, J.A., Schluter M. Fish provision in a changing environment: the buffering effect of regional trade networks. Submitted.

8. Folke C, Biggs R, Norström A V., Reyers B, Rockström J. Social-ecological resilience and biosphere-based sustainability science. Ecol Soc. 2016;21: 41. doi:10.5751/ES-08748-210341

9. Crona B, Nyström M, Folke C, Jiddawi N. Middlemen, a critical social-ecological link in coastal communities of Kenya and Zanzibar. Mar Policy. 2010;34: 761–771. doi:10.1016/j.marpol.2010.01.023

10. Drury O’Neill E. Catching values of small-scale fisheries: A look at markets, trade relations and fisher behaviour. Stockholm University. 2018.

11. Kluger LC, Scotti M, Vivar I, Wolff M. Specialization of fishers leads to greater impact of external disturbance: Evidence from a social-ecological network modelling exercise for Sechura Bay, northern Peru. Ocean Coast Manag. 2019;179: 104861. doi:10.1016/j.ocecoaman.2019.104861

12. Granovetter M. Economic Action and Social Structure: The Problem of Emeddedness. Am J Sociol. 1985;3: 69–83. doi:10.1086/228311

13. Clark CW. Mathematical bioeconomics: the mathematics of conservation (Vol. 91). John Wiley & Sons.; 2010.

14. R Core Team. R: A Language and Environment for Statistical Computing. Vienna, Australien: R Foundation for Statistical Computing; 2015. Available: https://www.R-project.org

15. Wilensky U, Stroup W. HubNet. http://ccl.northwestern.edu/netlogo/hubnet.html. Evanston, IL.; 1999.

16. Lindkvist E. FishMob: Fisher mobility and spatial resource heterogeneity: Patterns of overexploitation. CoMSES Computer Model Library; 2020. Available: https://www.comses.net/codebase-release/9e025075-af23-4acd-9fe2-18b4faba9c7c/

17. Munguia-Vega A, Weaver AH, Domínguez-Contreras JF, Peckham H. Multiple drivers behind mislabeling of fish from artisanal fisheries in La Paz, Mexico. PeerJ. 2021:e10750. Available from: https://peerj.com/articles/10750

1. Referred to as buyers in the netolog model. [↑](#footnote-ref-1)
2. The empirically-informed network files can be downloaded with the CoMSES package together with the netlogo file. [↑](#footnote-ref-2)
