# Supplementary material for: Fish provision in a changing environment: The buffering effect of regional trade networks
Source: PLoS One. 2021 Dec 20;16(12):e0261514. doi: 10.1371/journal.pone.0261514 (PMC8687593; doi:10.1371/journal.pone.0261514)
Supplement: S2 Appendix — (DOCX) [file pone.0261514.s002.docx]

**Supplementary information 2: Empirical analysis of interview data**

Table of Contents

[1. Quantitative analysis of survey questions to inform model design 1](#_Toc75332293)

[2. Qualitative analysis of interviews to inform the model processes 2](#_Toc75332294)

[References 5](#_Toc75332295)

# **1. Quantitative analysis of survey questions to inform model design**

The results of the closed-ended (i.e. survey) questions included in the semi-structured interviews are based on the analysis of interviews with 13 traders conducted in La Paz (Baja California Sur). These interviews were conducted in May-June 2019. Given the limited number of interviewees, these results only provide a qualitative understanding of patterns found in the study case.

Table A.1.1. shows the proportion of trade partners with whom a given trader considers to have a stable relationship. The results show that traders have stable relationships with about half or more than half of their trade partners. Traders were also asked about the nature of such relationships, asking with how many of their trading partners they have: written contracts, moral commitment, reciprocity, friendship, empathy, reliability, and information exchange. The results show that for most trading partners, those relations are characterized by moral commitment and other informal relationships rather than written contracts. Information exchange was particularly common as a characteristic of relationships between trade partners. These findings regarding the nature of trade relationships support findings described qualitatively in González-Mon et al. (2019).

Table A.1.2. shows the frequency at which traders report changing their trading relationships (e.g. trading with someone new or dropping relationships). Tables A.1.3. and A.1.4 provide further understanding on why traders would stop trading with one of their trade partners and what would they consider when starting a new relation. The current version of the Small-Trade model includes static networks based on the finding that relationships rarely change at a daily or weekly time-scale (Table A.1.2). The motivations for changing trading relations give insights about other processes that should be considered when adding new functionalities in the model that include trade network dynamics). To understand results in Table A.1.3, note that credit is often involved in these trading activities. Sellers would “loan” the fish and get the money 3-7 days later (usually to small shops or traders, and they have a reputation of paying on time or not).

**Table A.1.1. Self-identified stability of trade relationships.**

| How stable are your relationships? | N° respondents |
| --- | --- |
| With everyone | 1 |
| With most | **5** |
| With half | **5** |
| With a few | 2 |
| None | 0 |
| TOTAL | 13 |

**Table A.1.2. Changes in trade relationships.**

| How often do you change traders (stop buying or start selling with someone new)? | N° respondents |
| --- | --- |
| Every day | 0 |
| Every week | 1 |
| Every month | 3 |
| A few times a year (some state during a specific season, e.g., easter) | 3 |
| Less than once a year (every few years) | **5** |
| Few times a year | 1 |
| TOTAL | 13 |

**Table A.1.3. Stop a trade relationship.** Multiple choice items on reasons to stop trading with others.

| Reasons for stop buying/selling | N° respondents |
| --- | --- |
| Change prices | 2 |
| Cheat or do not hold their commitment | 2 |
| Problems with payments | **6** |
| Quality of the product | 2 |
| I never stop or it hasn't happened | 3 |

**Table A.1.4. Start a trade relationship.** Multiple choice items on reasons to start trading with others.

| Reasons for start buying/selling | N respondents |
| --- | --- |
| Generations or time working together | 2 |
| Personal relationship | 2 |
| Recommended through family or friends | 1 |
| Recommended through buyers | 2 |
| Price | 2 |
| Volume | **4** |
| Flexibility and payment | **5** |
| Constancy/evenness | 1 |
| Quality and legal origin | **3** |

#

# **2. Qualitative analysis of interviews to inform the model processes**

The model processes where informed by the 13 interviews conducted with traders in La Paz (Baja California Sur) in 2019. First, the qualitative interview data and its quantitative analysis (results above), together with the research conducted in González-Mon et al. (2019), informed the design of a first conceptual model (FigureS.1.1). The qualitative coding of the interviews is based on that conceptual model that provided general themes (e.g. fishing decisions, trade process) to enter the data analysis. Then, I coded the specific processes emerging from the data in sub-themes or codes following these general themes. **Table A.1.6.** shows the results of the qualitative analysis related to each of the steps in the model process. Note that the table includes some elements that have not been included in the ABM for simplicity, or that should be included in future model extensions (e.g. those marked with * in the table).


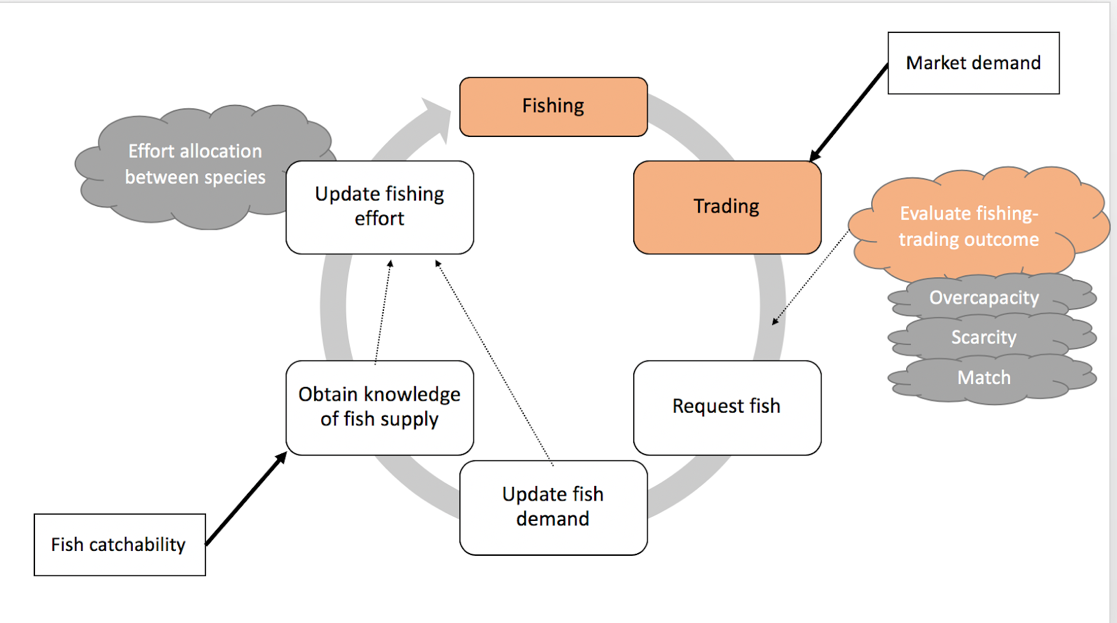


**FigureS.1.1. Conceptual model used to guide the thematic coding on the interviews**.

**Table A.1.6. Summary of themes and codes emerging from the qualitative data analysis.** General themes highlighted in bold, and underneath the codes that belong to each theme. Analysis performed in MAXQDA2020.

| **Themes and codes** | **Description** | **Model representation** |
| --- | --- | --- |
| **Fishing decisions** | **Traders perspective on how fishers decide what to fish and their role in affecting fisher’s effort** | **Activities 4-6 in table 2 in main paper** |
| Switch species | Fishers switch between species depending on their availability (e.g. seasons) and/or the market demand (e.g. if there is an oversupply of one species they can change to another one). In general, some traders report that “there is always something to fish” (trader 9, May 2019). Traders can also switch species in the event of scarcity (e.g. when the sand bass was banned, they traded mullet -both low value species-). | Effort allocation between species **(the main mechanism studied in this model).** |
| Switch location | Fishers move to fish in another location, chasing the fish or responding to seasons, or the trader facilitates the movements. Two interviewees stated that this strategy has decreased over the years due to regionalization policies and technological improvements (trader 14 and 17, May 2019). Alternatively, a trader can switch to buying from other locations temporarily. | Traders can buy from traders that get fish from other regions capturing this strategy at the trader’s level. *Not in the model at the fisher’s level /mobility (but see associated model in ComSES model library). |
| Fishers decide: availability and preferences | Fishers decide based on what there is in the water (what they can fish, what is “biting” or “pulling” at the moment), for example traders state that it is useless to tell fishers what to fish if the fish is not there. Also refers to fishers doing “whatever they want” and the trader having no role in what the fisher does. In that case it is related to fishers’ preferences. Fishers preferences in the case of La Paz/Gulf are often said to be to fish red snapper if it is biting (or higher value species). | Obtain knowledge of fish catch which is part of the CPUE function (they know how much they caught last week) + noise in the fishing effort to represent how much the fishers don't behave as the traders would. |
| Fishers decide: Keep fishing | Fishers keep fishing a commercially important species as long as possible regardless of the market signals (e.g., oversupply). It is often reported with seasonal high value species such as red snapper. | CPUE decision making model *Note this may imply a difference between HV and LV that is not implemented in the model. |
| Trader decides: Stop fishing | Traders can command fishers to stop fishing a certain species that they do not need, and/or stop buying it (e.g., oversupply). Linked to the “fishers decide” code, one strategy fishers can use to go about this command is keep fishing that species but in combination with others, to “force” traders to buy both species even if they only want one of them, therefore they do not completely stop fishing what they want (e.g., trader 10, May 2019). | Demand driven decision making model → when there is no demand of one species the function behaves so they reduce the effort to that species. |
| Trader decides: Fishing for demand | Traders report requesting certain species based on their current need / demand, and the fishers follow their request. | Demand driven decision making model. |
| **Trade strategy** |  | **Activities 1-3 and 7-9 in table 2 in main paper** |
| Assessing / knowing supply and demand | Traders report knowing who has the fish they need, knowing who to ask if they need fish. They know this because they are in communication with the cooperative/producers to know how much fish is going to come and they report calling clients to know how much fish they need. Some also say that there are “rumors” to know who has fish, and that everyone knows each other after many years trading fish. | Update demand  Availability of fish per actors is known by everyone. |
| Dealer’s mechanism | Some “patrons” buy fish besides their fishers to satisfy their demand. Dealers can also sell to each other to get the fish they need since different actors may have different species/amounts at times, or as some kind of assistance or reciprocity (e.g., selling fish to get bait). | Horizontal trade, dealers can request fish. |
| Sellers offer fish | Traders report calling each other to offer fish. Mainly sellers call offering the fish they have and for some this would especially happen when there are market gluts (but also happens as part of the normal activities). | Continue-trading step. |
| Buyers request fish | Traders report calling each other to request what they need. | Request mechanism. |
| Highest bidder | Important for some to choose partners and try to change partners. There can be cheating to sell a higher bidder at times but not necessarily breaking completely the commitments. Some say it's better that they pay good (on time, not the highest) and that it is not worth it to try to sell/buy fish for 1-3MXN more/less since it is better to sell/buy to the people you already trust. | *Selling outside the stable trade network is not implemented in the model |
| **Trading outcomes-actions** | Situations or changes when supply/demand are not on equilibrium. | **Assumptions and scenarios** |
| Overcapacity | Traders describe situations or market gluts, when they sell their catch to new markets, more buyers, or lower the price. Alternatively, they can also stop “producing” fish if they have fishers attached or stop buying fish. Sellers can also switch species. Those that have the capacity to freeze or transform the fresh fish would do so. In addition, there is a mechanism or help or prioritize, where traders keep buying their stable relationships in case of gluts (even a small quantity if they don't need). | Lim_trade_dmd ON → Waste  *Stop buying or lower price is implemented now as no income for seller for the “oversupply” when dmd<catch. Freezing or transforming is not in the model. Looking for alternative buyers is not in the model. All that is captured under the “waste” variable. |
| Scarcity | Fish scarcity is reported as a seasonal phenomenon or related to some extreme natural events such as red tides or hurricanes. In those situations, traders report getting fish from somewhere else (buyers even import in some cases) or getting other species. Some would just stop their fishing activity until times get better. In addition, some give priority for buying/selling from actors with committed relationships. | This happens as a consequence of model experiments (e.g., catchability 0) and traders can trade with their committed partners to get fish.  *Stop buying fish or fishing is not possible in the model |

# **References**

González-Mon B, Bodin Ö, Crona B, Nenadovic M, Basurto X. Small-scale fish buyers’ trade networks reveal diverse actor types and differential adaptive capacities. Ecol Econ. 2019;164: 106338. doi:10.1016/j.ecolecon.2019.05.018
