# Supplementary material for: Fish provision in a changing environment: The buffering effect of regional trade networks
Source: PLoS One. 2021 Dec 20;16(12):e0261514. doi: 10.1371/journal.pone.0261514 (PMC8687593; doi:10.1371/journal.pone.0261514)
Supplement: S3 Appendix — (DOCX) [file pone.0261514.s003.docx]

**Supplementary information 3: Network analysis and generation of experiments for the ABM**

Table of Contents

[1. Data collection 1](#_Toc75332363)

[2. Analysis of the empirical network 2](#_Toc75332364)

[3. Results 2](#_Toc75332365)

[4. Generating networks for ABM experiments 4](#_Toc75332366)

[References 5](#_Toc75332367)

The model scenarios are based on empirically-informed network structures. Below we further explain the method used to generate such structures and show the results of the Exponential Random Graph Modelling (ERGM) method referred to in the main manuscript.

# **1. Data collection**

The data used to generate network scenarios was collected in González-Mon et al. (2019). Their study used a snowball sampling technique to identify all actors that trade fish from the southern Corredor region (comprising 9 fishing communities) to and within the city of La Paz in Baja California Sur. In addition, a survey to 52 fishers (70-75% of the boat captains in the region) asking to whom do they sell their catch was used to identify traders, and map and understand their relationship with fishers. These processes resulted in the identification of 28 traders. Semi-structured interviews and participant observation methods were conducted with 23 of these traders (81%) asking to whom to they buy/sell their fish and the frequency of those relationships to build the trade network that is used in this study. In addition, questions were also asked to understand the nature of the relationships and trading activities, which was used in a qualitative analysis as reported in González-Mon et al. 2019. Please refer to the cited study for more details on the data collection process.

For the network used in this manuscript, we took a subset of the network published in González-Mon et al. (2019). This subset consisted of selecting the traders that had direct relationships to the fishers (and therefore operating as “patrons”), because in this study we aim to investigate the role of relationships within the same level of traders. This excluded 5 exporters/intermediaries and 3 municipal markets will all their associated relationships. González-Mon et al. (2019) also differentiates between two types of relationships, regular relationships that occur with a given frequency (weekly, monthly or seasonally) and infrequent relationships that occur occasionally or less than once a month. For this study we decide to include only regular relationships, since those are the ones that most likely represent stable relationships do not change between years (this implies deleting 4 relationships since most were regular).

In addition, while collecting the data to build the traders network in González-Mon et al. (2019), they identified important characteristics of the traders through the qualitative descriptions in semi-structured interviews. We did not have complete information of all characteristics for all 15 traders, and we only consider as potential attributes to use as input for the ERGM analysis the ones with information for the 15 traders (see below).

# **2. Analysis of the empirical network**

We first analyze the network using Exponential Random Graph Modelling (ERGM) in Mpnet, starting from the findings reported in González-Mon et al. (2019). González-Mon et al. (2019) identified two different types of patrons (exchangers/dealers and suppliers) with different roles in the network, and link these types with the connections to the markets. González-Mon et al. (2019) also suggest that exchangers/dealers can have warehouses, be linked to fishers in several fishing communities, and sell to some final markets themselves without the need for other intermediaries, as opposed to suppliers and fishers that act as suppliers. Therefore, for this analysis we add a binary attribute (0/1), where 1 represents actors with direct links with external markets (who are the same actors that have a fish plant or warehouse in the city), vs. those that only sell to others in the city and do not have a warehouse (i.e. those selling in internal markets). We hypothesize that this attribute is a characteristic that can explain the differences between traders previously identified to some extent. Theoretically, we can also hypothesize that actors with higher physical/economic capacities or assets such as higher storage capacities and links to external markets, would be able to buy fish from others and have a more central role in the network.

Estimating the parameters or coefficients of an ERGM requires specifying the configurations that are expected to influence the formation of a given network structure. For the BCS trade network, this included density (ArcA), reciprocity (ReciprocityA), the centralization of buying and selling relationships (In2StarA, Out2StarA), and the number of traders without connections (isolateA). Then we tested two alternative hypotheses. One where the traders’ attribute was important to explain the network, and therefore the existence of this attribute would influence trader’s propensity to form buying relationships with others as mentioned above. And another hypothesis, where the structural processes alone (without any attribute) are able to represent the empirical network structure. We name the model with all parameters plus the traders’ attribute ERGM_1_, and the model without the traders’ attribute ERGM_2_ (Table A.2.1.).

# **3. Results**

Estimating the network without traders’ attributes (ERGM_2,_ Table A.2.1) seems to generate a model with a good fit for the parameters included (ERGM_2,_ Table A.2.2), but it doesn’t capture the role of trader types that we identified in our previous work as we see below (Table A.2.1in red). If we observe the types of networks generated, many networks will consist of networks with a majority of traders that can buy fish and we would categorize as dealers/exchangers, whereas in the empirical sample those type of actors are a minority (González-Mon et al. 2019). When we account for the attributes in the ERGM (ERGM_1,_ Table A.2.2), it is able to better reproduce the empirical patterns associated with the types of actors we found empirically (compare ERGM_1_ and ERGM_2_ in Table A.2.2,). Amongst other things, such as the visual exploration of the characteristics of the networks generated, this is evidenced by the higher Mahalanobis distance in ERGM_2_ (Table A.2.2). Besides the network characteristics, when we observe the networks generated, ERGM_1_ will mostly generate networks with about 5-6 traders that we would categorize as dealers which is closer to our analysis of the empirical network than in the model without attributes (ERGM_2_). Therefore, considering attributes of the traders may be key to explain the empirical patterns found in the trade network. This also means that some characteristics of the actors can be key in determining their role in the network (and not structural processes alone). Specifically, access to markets and having physical assets such as storage capacity may be key factors explaining the differential role of traders in this network, even if we cannot conclude that this are determining factors since we did not account for other potential characteristics that may create similar differences between traders.

**Table A.2.1.** Configurations and estimated parameters (coefficients) of the two ERGMs used to generate the two network structures used as experiments in the ABM. * Indicates significantly different than expected by chance.

| Model | configurations | Lambda | parameter | stdderr | t-ratio | sacf |
| --- | --- | --- | --- | --- | --- | --- |
| ERGM_1_ | **ArcA** | 2.0000 | -6.4769 | 1.624 | 0.051 | 0.779  * |
|  | **ReciprocityA** | 2.0000 | 5.2857 | 2.168 | 0.039 | 0.854  * |
|  | **In2starA** | 2.0000 | 0.0533 | 0.143 | 0.036 | 0.759 |
|  | **out2starA** | 2.0000 | 0.3869 | 0.269 | 0.064 | 0.756 |
|  | **IsolateA** | 2.0000 | -0.0779 | 1.254 | 0.001 | 0.300 |
|  | **SenderA** | 2.0000 | -2.1155 | 1.158 | 0.037 | 0.918 |
|  | **ReceiverA** | 2.0000 | 4.8769 | 1.561 | 0.035 | 0.759  * |
| ERGM2 | **ArcA** | 2.0000 | -3.0407 | 0.563 | 0.062 | 0.892 * |
|  | **ReciprocityA** | 2.0000 | 1.9250 | 0.825 | 0.059 | 0.745 * |
|  | **In2starA** | 2.0000 | 0.4010 | 0.055 | 0.053 | 0.745 * |
|  | **out2starA** | 2.0000 | -0.1133 | 0.217 | 0.066 | 0.843 |
|  | **IsolateA** | 2.0000 | 0.4491 | 0.972 | -0.085 | 0.731 |

**Table A.2.2. Goodness Of Fit (GOF) results for the two alternative ERGMs as compared to the empirical network (observed).** Simulated networks from the ERGMs showed as the mean across 10000 networks (mean); t-ratio = (observation - sample mean)/standard error; StdDev, Standard deviation. #, indicates values where the mean of the simulated networks differs substantially from the empirical network.

| MODEL | STATISTICS/CONFIGURATIONS | OBSERVED | MEAN | STDDEV | T-RATIO |
| --- | --- | --- | --- | --- | --- |
| ERGM_2_ - without attributes in the model estimation | **ArcA** | 26.0000 | 25.077 | 13.876 | 0.067 |
|  | **ReciprocityA** | 5.0000 | 4.7330 | 3.845 | 0.069 |
|  | **In2starA** | 58.0000 | 54.6030 | 63.013 | 0.054 |
|  | **out2starA** | 24.0000 | 22.4010 | 26.332 | 0.061 |
|  | **ATA-D** | 12.0000 | 11.2634 | 15.506 | 0.048 |
|  | **ATA-U** | 16.5000 | 15.2285 | 22.936 | 0.055 |
|  | **IsolateA** | 2.0000 | 2.1090 | 2.326 | -0.047 |
|  | **Externalmarket_senderaA** | 9.0000 | 8.3560 | 5.077 | 0.127 |
|  | **Externalmarket_receiverA** | **24.0000** | **8.8420** | **6.953** | **2.180 #** |
|  | **Externalmarket_interactionA** | **8.0000** | **2.4990** | **2.504** | **2.197 #** |
|  | **Internalmarket_senderA** | 17.0000 | 16.7210 | 9.381 | 0.030 |
|  | **Internalmarket_receiverA** | **2.0000** | **16.2350** | **9.872** | **1.442** |
|  | **Internalmarket_interactionA** | **1.0000** | **10.3780** | **6.798** | **-1.380** |
|  | **Mahalanobis distance = 283**  **Maximum qasi-autocorrelation in absolute value = 26890.014** | | | | |
| ERGM_1_ - with attributes in the model estimation | **ArcA** | 26.0000 | 25.8240 | 8.231 | 0.021 |
|  | **ReciprocityA** | 5.0000 | 4.8480 | 3.041 | 0.050 |
|  | **In2starA** | 58.0000 | 57.4470 | 4.551 | 0.016 |
|  | **out2starA** | 24.0000 | 23.5010 | 5.051 | 0.033 |
|  | **ATA-D** | 12.0000 | 11.8742 | 10.222 | 0.012 |
|  | **ATA-U** | 16.5000 | 15.6192 | 14.579 | 0.060 |
|  | **IsolateA** | 2.0000 | 1.9660 | 1.387 | 0.025 |
|  | **Externalmarket_senderaA** | 9.0000 | 8.7810 | 5.003 | 0.044 |
|  | **Externalmarket_receiverA** | 24.0000 | 23.9760 | 7.210 | 0.003 |
|  | **Externalmarket_interactionA** | 8.0000 | 7.4220 | 7.4220 | 0.138 |
|  | **Internalmarket_senderA** | 17.0000 | 17.0430 | 4.896 | -0.009 |
|  | **Internalmarket_receiverA** | 2.0000 | 1.8480 | 1.763 | 0.086 |
|  | **Internalmarket_interactionA** | 1.0000 | 0.4890 | 0.854 | 0.598 |
|  | **Mahalanobis distance = 53**  **Maximum qasi-autocorrelation in absolute value = 1815.904** | | | | |
|  |  | | | | |

Besides this overarching result comparing the two network models qualitatively, the statistical results of the model estimation (Table A.2.1) show that there is a significant tendency of actors with links to external markets and a warehouse to buy fish from others as compared to traders without those characteristics. There is also a significant tendency to have reciprocal links, meaning that likely those actors are buying and selling fish to each other (i.e. “exchanging”) more than expected by chance. This coincides with our proposition in González-Mon et al. (2019) that there is a type of patron that both buys and sells fish from/to others and that we hypothesize have higher capacity to adapt to changes.

# **4. Generating networks for ABM experiments**

ERGM can be seen as a tool to generate random networks with particular attention to specific network characteristics. In this way we simulate random networks based on the characteristics of the empirical trade network, by specifying different hypotheses that can lead to trade network structures, that are then used as experiments in the ABM (Figure S.2.1). The model with the best fit (ERGM_1_, Figure S.2.1) generates networks with similar characteristics than the empirical network, of which we selected one for one of the ABM model experiments. We can also use the alternative hypothesis presented above as one model experiment (ERGM_2_), to understand the influence of this alternative hypothesis on model outcomes. In this case, the experiment implies changing the processes that are on the first place assumed to influence the network structure, and thus getting a hypothetical scenario that shows some of the characteristics of the empirical network but that differ in one critical process, which in this case was removing the influence of individual attributes on the network (ERGM_2_ in Figure S.2.1). Therefore, building ERGMs with different configurations that explain the network is one way to generate or simulate counterfactual networks that can be used as input in an ABM as we implement in this manuscript. Alternatively, parameters of an ERGM can be modified to increase or decrease the tendencies to form specific configurations and then generate different networks based on such counterfactual hypothesis with the same configurations but different parameters (González-Mon 2020).

The ERGM_1_ can be used to simulate networks that approximate the characteristics of the empirical network, as shown by the Goodness Of Fit (Table A.2.2). From this analysis, we obtain networks that fill the possibility space of all networks that could be generated under the mechanism that we hypothesize in our ERGM. This can be used to select networks similar to our empirical network, since in fact the networks generated concentrate around the values of the empirical network. The “empirically-simulated” network experiment is one of the networks that has similar characteristics to the empirical network generated with the ERGM_1_ model (Table A.2.3). Similarly, the “more-dealers” network in the manuscript (Figure S.2.1) was selected amongst the networks that were simulated with the ERGM_2_ model, choosing a network with similar characteristics as the empirical network amongst all simulated networks (Table A.2.3), but with different proportion of trader types. Given that not all networks generated with the ERGMs have the same characteristics of the empirical network, we tried to select networks that resemble the empirical networks in key characteristics such as density, clustering coefficient (transitivity), and degree distribution; and report those values to aid interpretation noting that not all values exactly correspond to those of the empirical network (Table A.2.3). We also observe other networks along the spectrum of characteristics of the possibility space generated with the ERGMs that differ substantially from the empirical network. Different networks within the possibility spaces could also be used as model experiments but are not considered in this manuscript.

**Table A.2.3. Characteristics of the networks used as experiments as compared to the empirical network with 16 nodes**. Degree distribution represented through the mean, standard deviation and skewness of degree values in the network (accounting for both in and out degree). Density, clustering and degree measures calculated with the Igraph package in R.

| NETWORK | DENSITY | CLUSTERING COEFFICIENT | MEAN DEGREE | STDDEV DEGREE | SKEWNESS DEGREE |
| --- | --- | --- | --- | --- | --- |
| Empirical | 0.108 | 0.284 | 3.25 | 3.296 | 0.228 |
| Empirically-simulated | 0.086 | 0.333 | 2.625 | 2.604 | 1.296 |
| More-dealers | 0.108 | 0.317 | 3.25 | 2.671 | 0.842 |


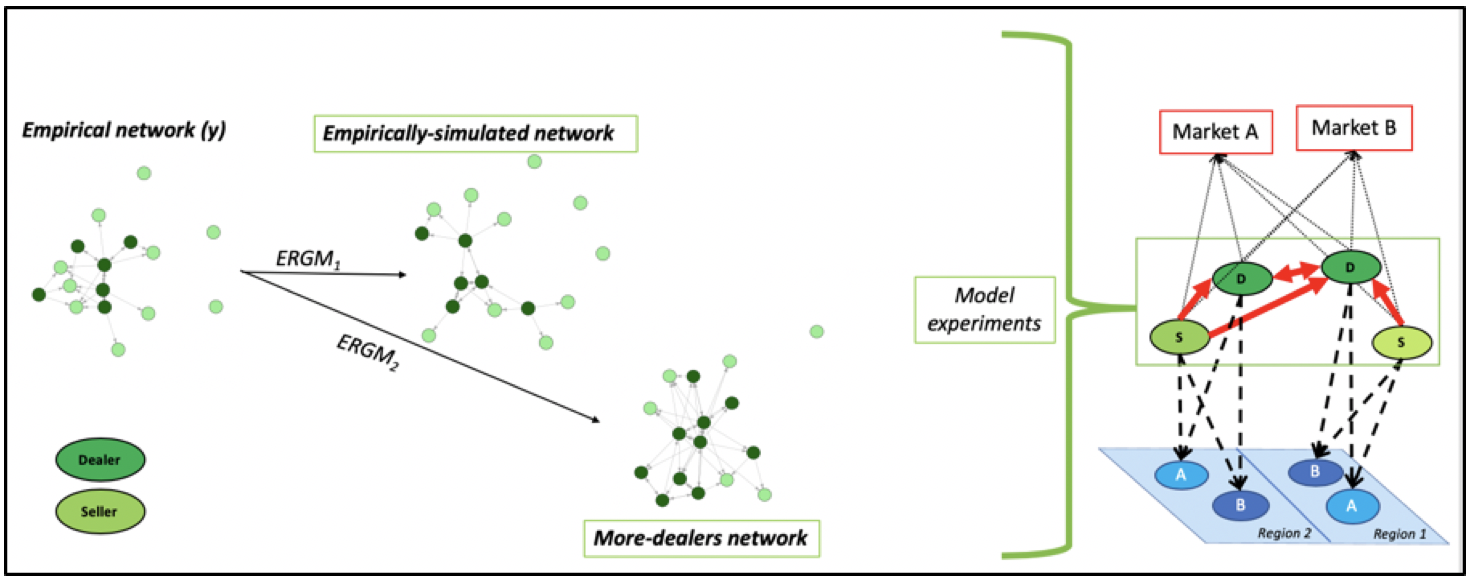


**Figure S.2.1. Simulating empirically-informed trade networks and their integration in the Small-Trade model.** We simulated 2 trade networks using Exponential Random Graph Modelling (ERGM). ERGM_1_ simulated a network that tries to resemble the empirical trade network. ERGM_2_ removed one of the network-formation mechanisms that explain the trade network, generating an alternative network with different proportion of trader types. The 2 different network structures are used as experiments in the Agent-Based Model. Note that directionality of the links (D🡪S) implies D “request fish to” and “buy from” S (which is the directionality used in the ABM, but opposite (transposed) to the empirical networks used in the ERGM). Figure and caption modified from González-Mon 2020.

# **References**

González-Mon B, Bodin Ö, Crona B, Nenadovic M, Basurto X. Small-scale fish buyers’ trade networks reveal diverse actor types and differential adaptive capacities. Ecol Econ. 2019;164: 106338. doi:10.1016/j.ecolecon.2019.05.018

González-Mon, B. Cross-scale mechanisms and adaptation strategies in Small-Scale Fisheries. Licentiate thesis, Stockholm University. 2020. Retrieved from: https://www.diva-portal.org/smash/record.jsf?pid=diva2:1470226
