# Supplementary material for: Fish provision in a changing environment: The buffering effect of regional trade networks
Source: PLoS One. 2021 Dec 20;16(12):e0261514. doi: 10.1371/journal.pone.0261514 (PMC8687593; doi:10.1371/journal.pone.0261514)
Supplement: S4 Appendix — (DOCX) [file pone.0261514.s004.docx]

**Supplementary information 4: Sensitivity analysis**

Table of Contents

[1. Analysis of the influence of trader types 1](#_Toc75418121)

[**Influence at the market’s level (Fig S4.1)** 2](#_Toc75418122)

[**Influence on overexploitation (Fig S4.2)** 2](#_Toc75418123)

[**Influence at the trader’s level (Fig S4.3)** 3](#_Toc75418124)

[2. Analysis of the influence of network density 3](#_Toc75418125)

[**Influence at the market’s level (Fig S4.4 and S4.5)** 4](#_Toc75418126)

[**Influence on overexploitation (Fig S4.6)** 5](#_Toc75418127)

[**Influence at the trader’s level (Fig S4.7)** 5](#_Toc75418128)

[3. Analysis of the decision-making model with different balance rates 6](#_Toc75418129)

[**Influence at the market’s level (Fig S4.8)** 6](#_Toc75418130)

[**Influence on overexploitation (Fig S4.9)** 7](#_Toc75418131)

[**Influence at the trader’s level (Fig S4.10)** 8](#_Toc75418132)

[4. Non-aggregated time series of all network structures 8](#_Toc75418133)

[Scenario 1. Seasonality (Fig S4.11 and S4.12) 9](#_Toc75418134)

[Scenario 2. Catch variability (Fig S4.13 and S4.14) 11](#_Toc75418135)

# **1. Analysis of the influence of trader types**

This sensitivity analysis tests the influence of the trader types (proportion of sellers), keeping other parameters in the model as constant. The experiments vary the proportion of sellers (where 1=all sellers and 0=all dealers) in a random network with *p*=1 (where *p* indicates the probability of each trader to create a link with others). Thus the networks consist on dealers that can buy from all other traders, and sellers that can sell to all the dealers in the network.

### **Influence at the market’s level (Fig S4.1)**

**
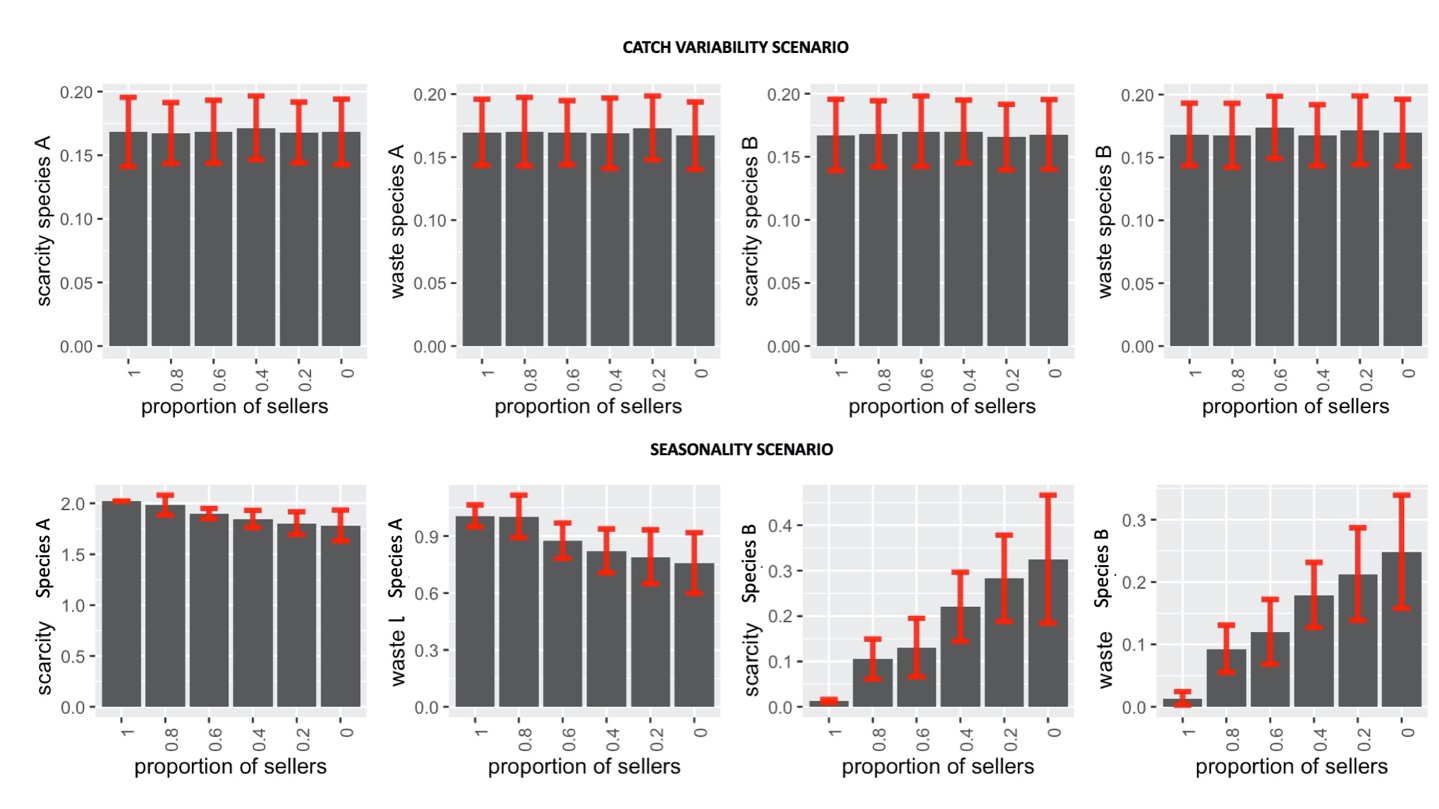
**

**Fig S4.1. Average and variability of scarcity and waste.** Top panels, catch variability scenario; bottom panels, seasonality scenario (seasonality species A in region 1). Average values across 50 runs.

### **Influence on overexploitation (Fig S4.2)**

**
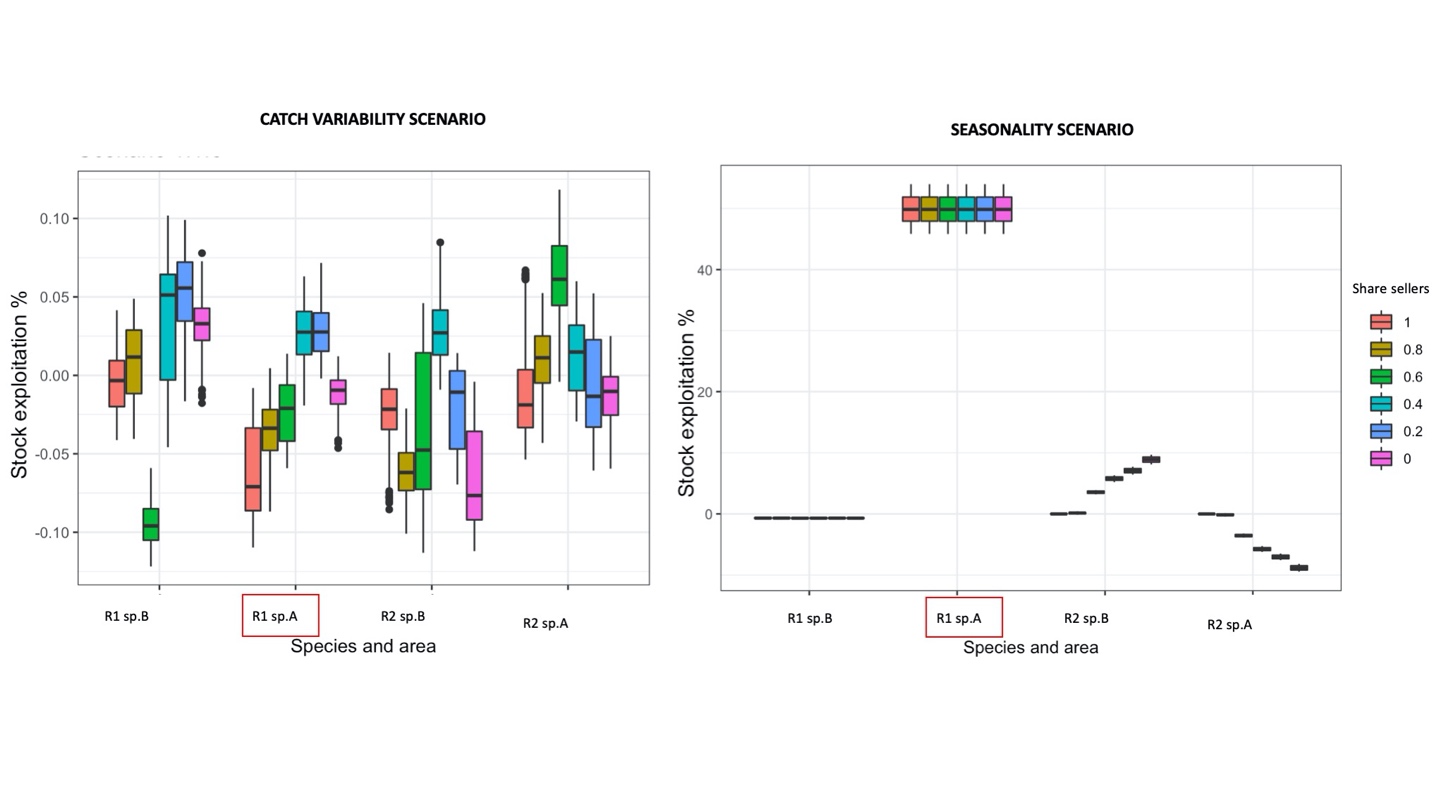
Fig S4.2. Average exploitation level of the 4 fish stocks.** Left panel, catch variability scenario; right panel, seasonality scenario (seasonality species A in region 1). Colors indicate networks with different proportion of sellers, where 0 (pink) indicates all dealers and 1 (red/orange) all sellers. Average values across 50 runs.

### **Influence at the trader’s level (Fig S4.3)**

**
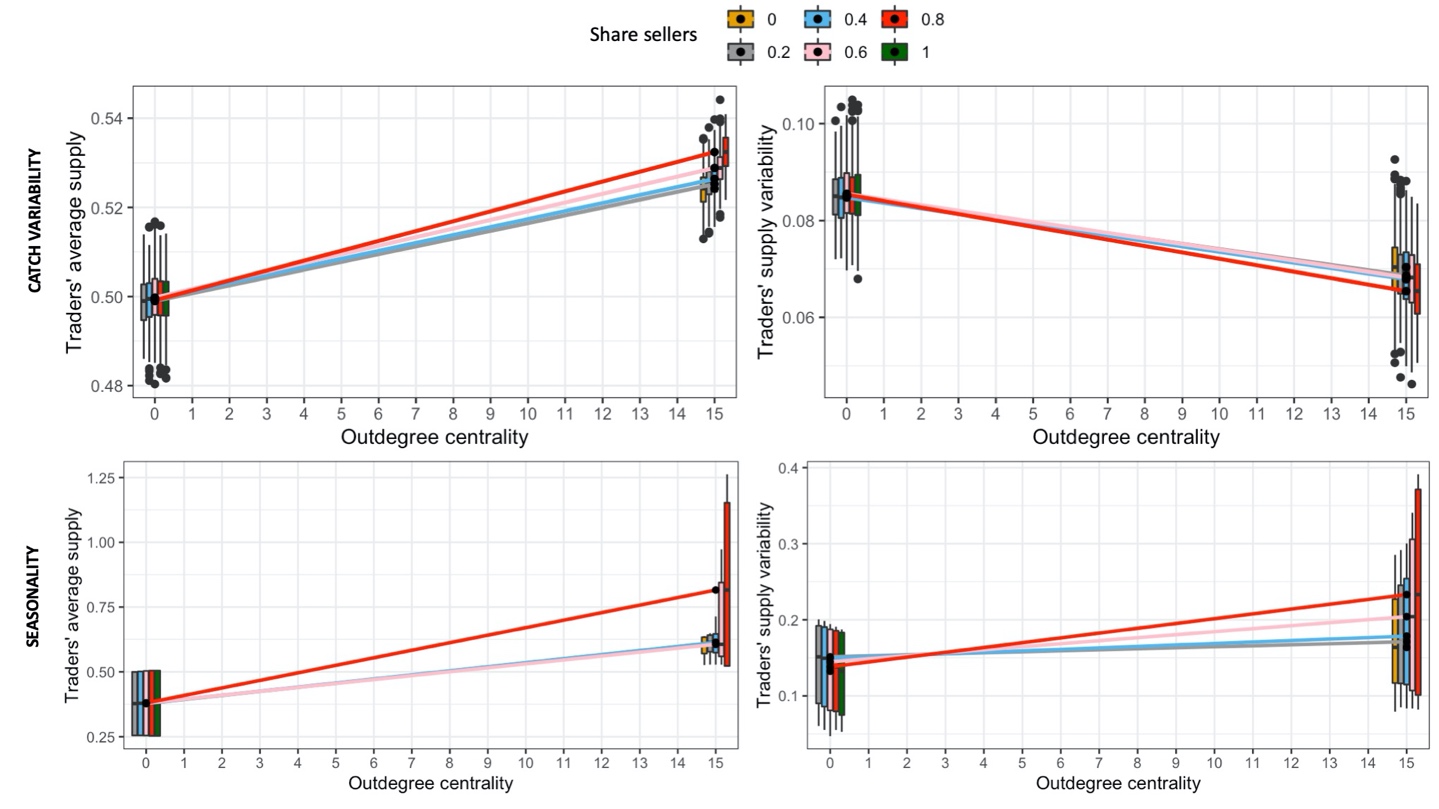
**

**Fig S4.3**. Box and whisker plots indicating the average and variability of fish supply (considering both species types) as a function of traders’ outdegree centrality, in response to: Top panels, catch variability scenario; bottom panels, seasonality scenario (seasonality species A in region 1). Colors indicate the proportion of sellers, where 0 (yellow) indicates all dealers and 1 (green) all sellers. Each box represents the outcomes for each trader in 50 simulations for each proportion of sellers, grouped based on their outdegree centrality. Since the networks are fully connected, all dealers have 15 out-links. All sellers have 0 out-links by definition of the trader type.

# **2. Analysis of the influence of network density**

This sensitivity analysis tests the influence of the network density (number of links), keeping other parameters in the model as constant. The experiments vary the density (*p*=0.2, 0.4, 0.6, 0.8, 1) in a random network and where all traders are dealers. *p* is the probability of having a link in the network (density) and it is measured in the plots below as the number of links in the network or as *p*, but both values are related since the exact number of links will be related to the probability of creating a link.

### **Influence at the market’s level (Fig S4.4 and S4.5)**

**
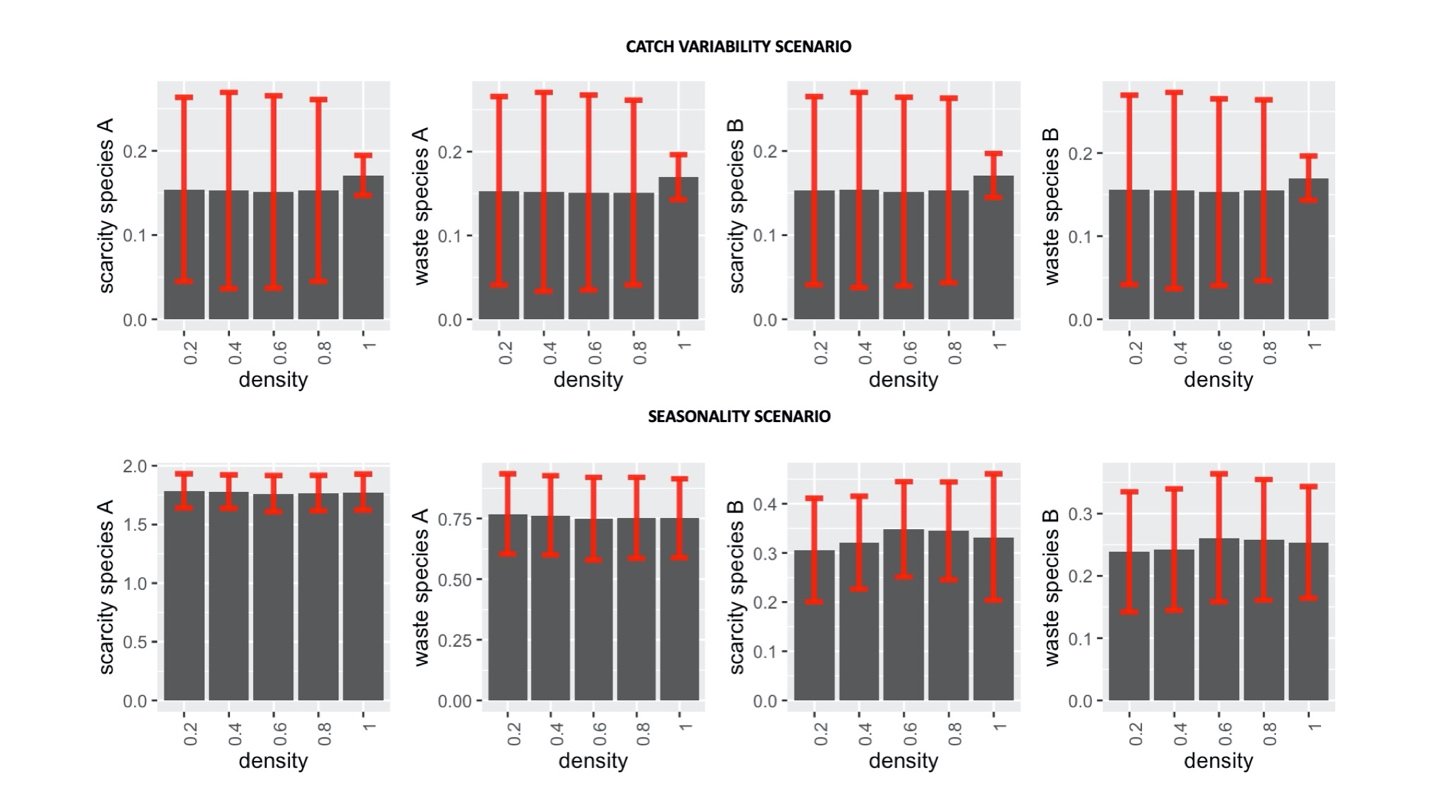
**

**Fig S4.4**. Average and variability of scarcity and waste. Top panels, catch variability scenario; bottom panels, seasonality scenario (seasonality species A in region). Density = probability of creating a link in the network. Average values across 50 runs.


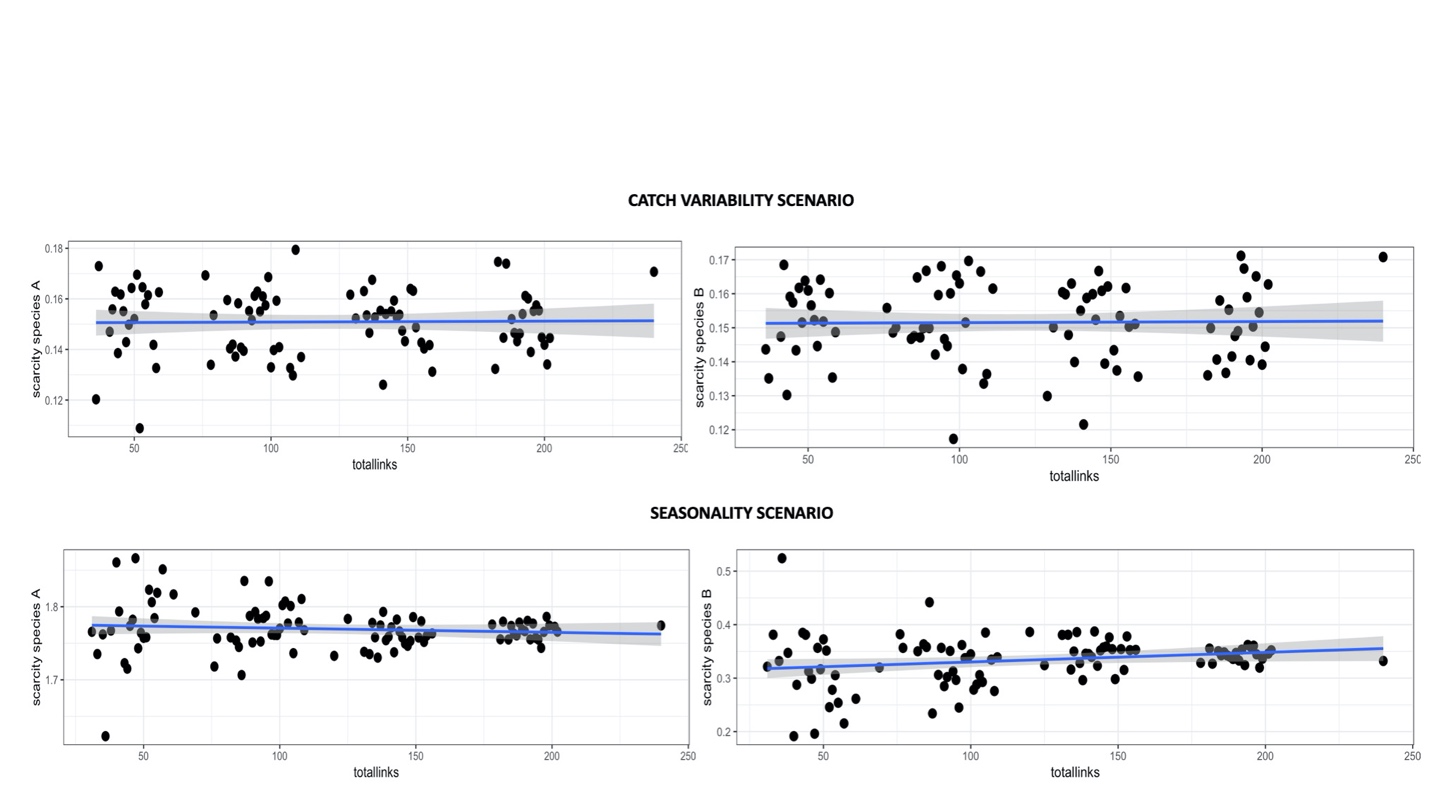


**Fig S4.5. Scatterplots of the average scarcity as a function of the number of links in the networks.** Top panels, catch variability scenario; bottom panels, seasonality scenario (seasonality species A in region). Total links indicate the number of links in the network, but note that in Netlogo a bi-directional or reciprocal link is counted as 2 links (out-link + in-link), and therefore the density value shown here could be divided to obtain the number of reciprocal links as usually measured in empirical network analyses. Each dot represents the average scarcity produced with each network generated in one of the 250 simulations run with density varying as indicated above. This Figure is intended to aid in the interpretation of Fig S.4.4., where we do not observe a strong influence of density. Note that networks with density=1 or 240 links are fully connected networks of dealers as the one presented in the main paper.

### **Influence on overexploitation (Fig S4.6)**

**
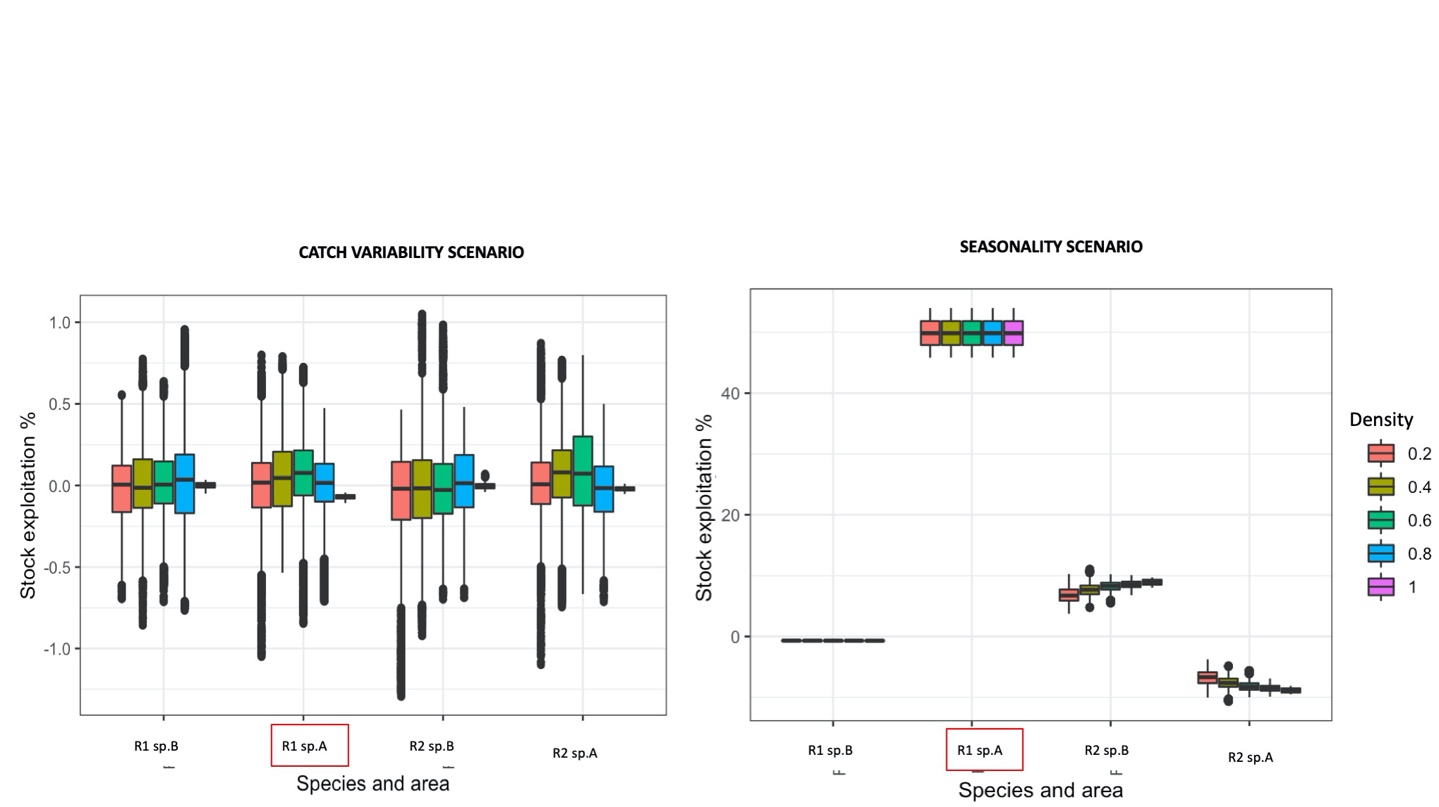
**

**Fig S4.6.** Average exploitation level. Left panel, catch variability scenario; right panel, seasonality scenario (seasonality species A in region). Density = probability of creating a link in the network as in Fig S.4.4.

### **Influence at the trader’s level (Fig S4.7)**


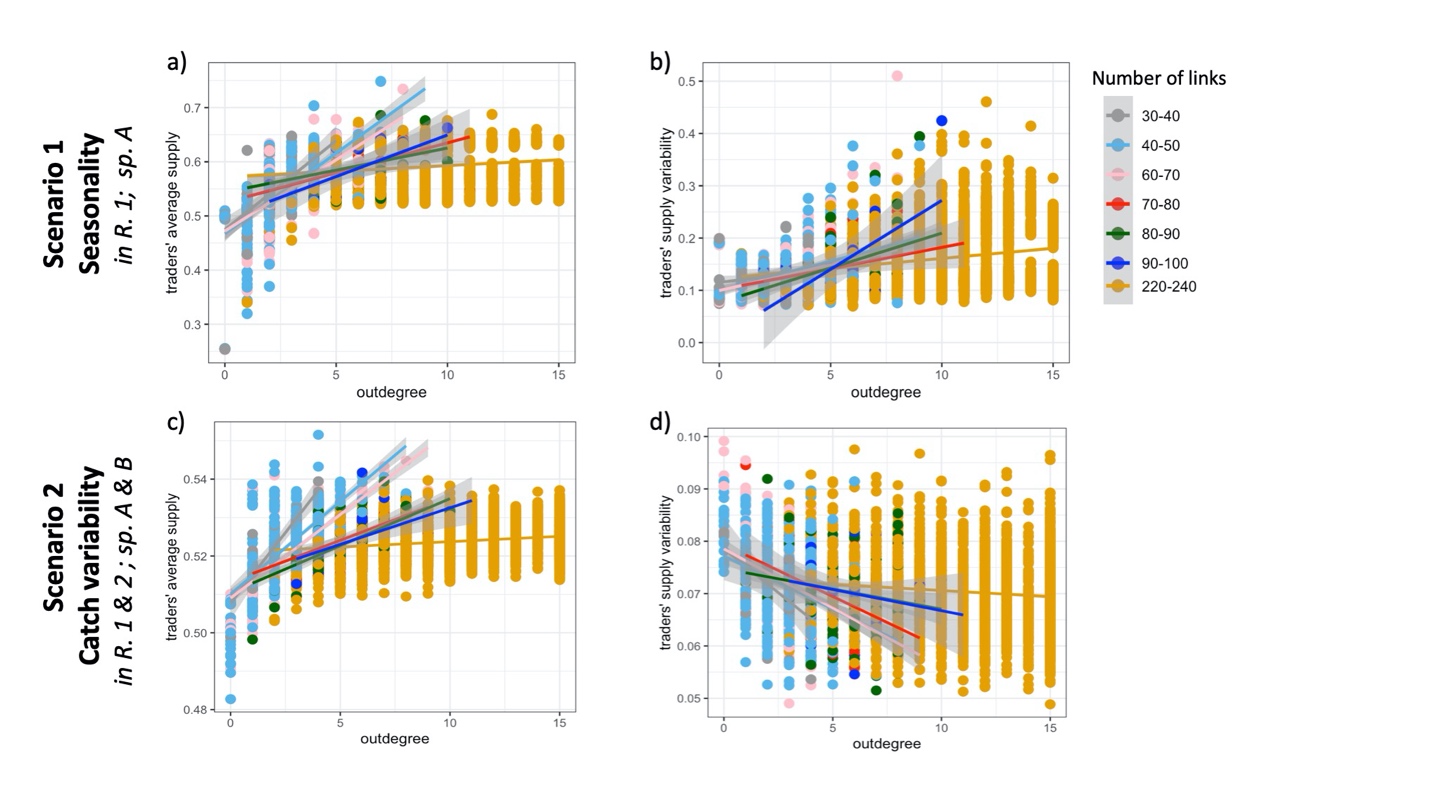


**Fig S4.7.** Scatter plots of the average (median) and variability (standard deviation) of fish supply (considering both species types) at the trader level as a function to trader´s outdegree centrality, in response to: Top panels, seasonality scenario (seasonality species A in region); bottom panels, catch variability scenario. Data points represent each of the 16 traders in 50 runs per density level (probability of creating a link as indicated above). Each data point is colored according to the number of links of the network they belong to, where networks with similar number of links were grouped in discrete bins for better visualization. Trends were estimated with a linear model.

# **3. Analysis of the decision-making model with different balance rates**

This sensitivity analysis tests the effect of the decision-making model selected in the main paper, where the balance rate (br) is set to “0.5”. The balance rate indicates the gradient between demand-driven (br=0) and CPUE-driven (br=1) decision making models. For this sensitivity analysis, the proportion of sellers is set to 0.6 (60% sellers/40%dealers), which is similar to the empirical network, and the test conducted in a random network with *p*=1 (therefore similar setting as the sensitivity analysis that tests the proportion of sellers). The switch rate, which determines how much can traders switch their effort from one species to the other in one-time step, is set at a maximum of 20% change as in the manuscript experiments.

### **Influence at the market’s level (Fig S4.8)**


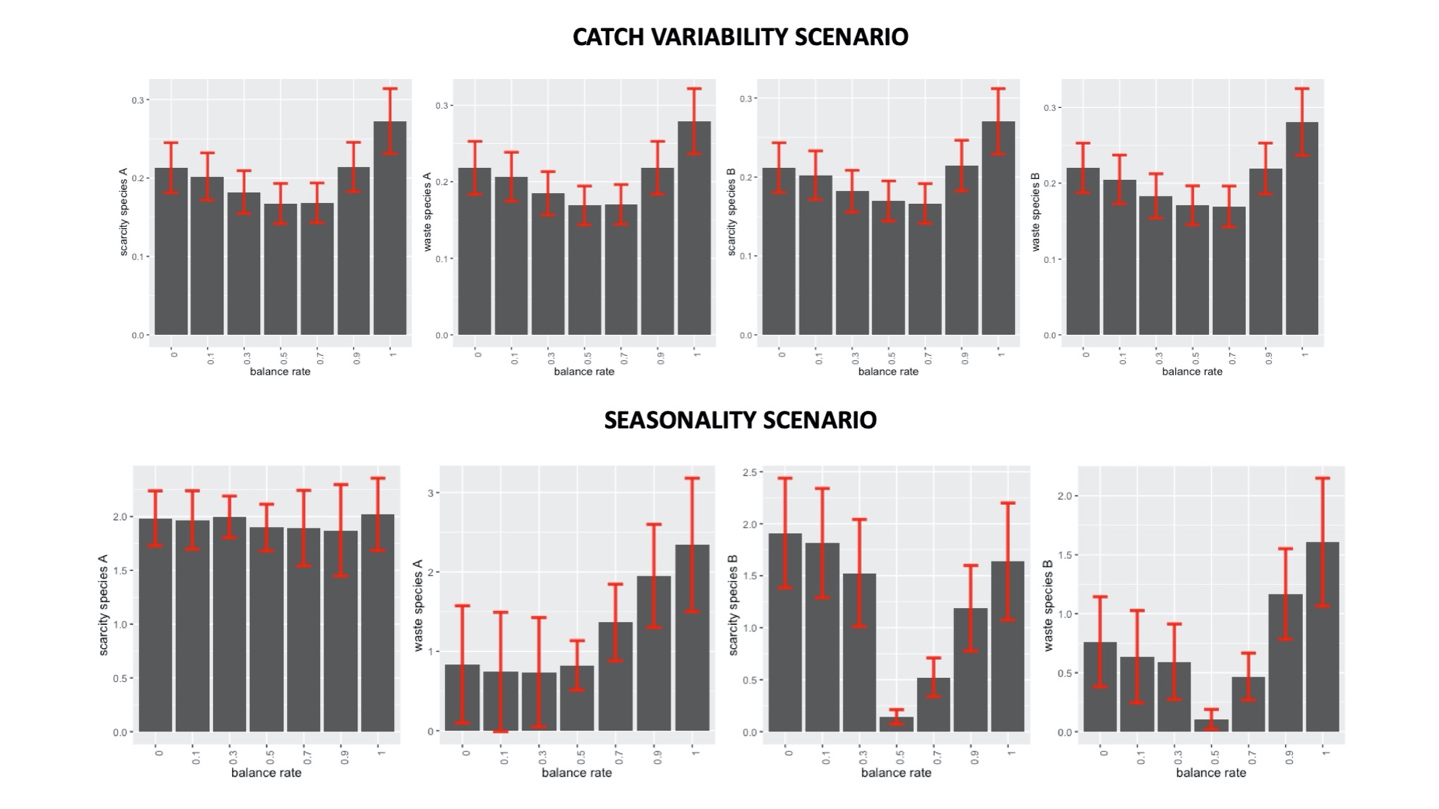


**Fig S4.8**. Average and variability of scarcity and waste. Top panels, catch variability scenario in both regions; bottom panels, seasonality scenario (seasonality species A in region 1). Balance rate indicates the decision-making model, where demand-driven=0; and CPUE-driven=1. Average across 50 runs.

### **Influence on overexploitation (Fig S4.9)**


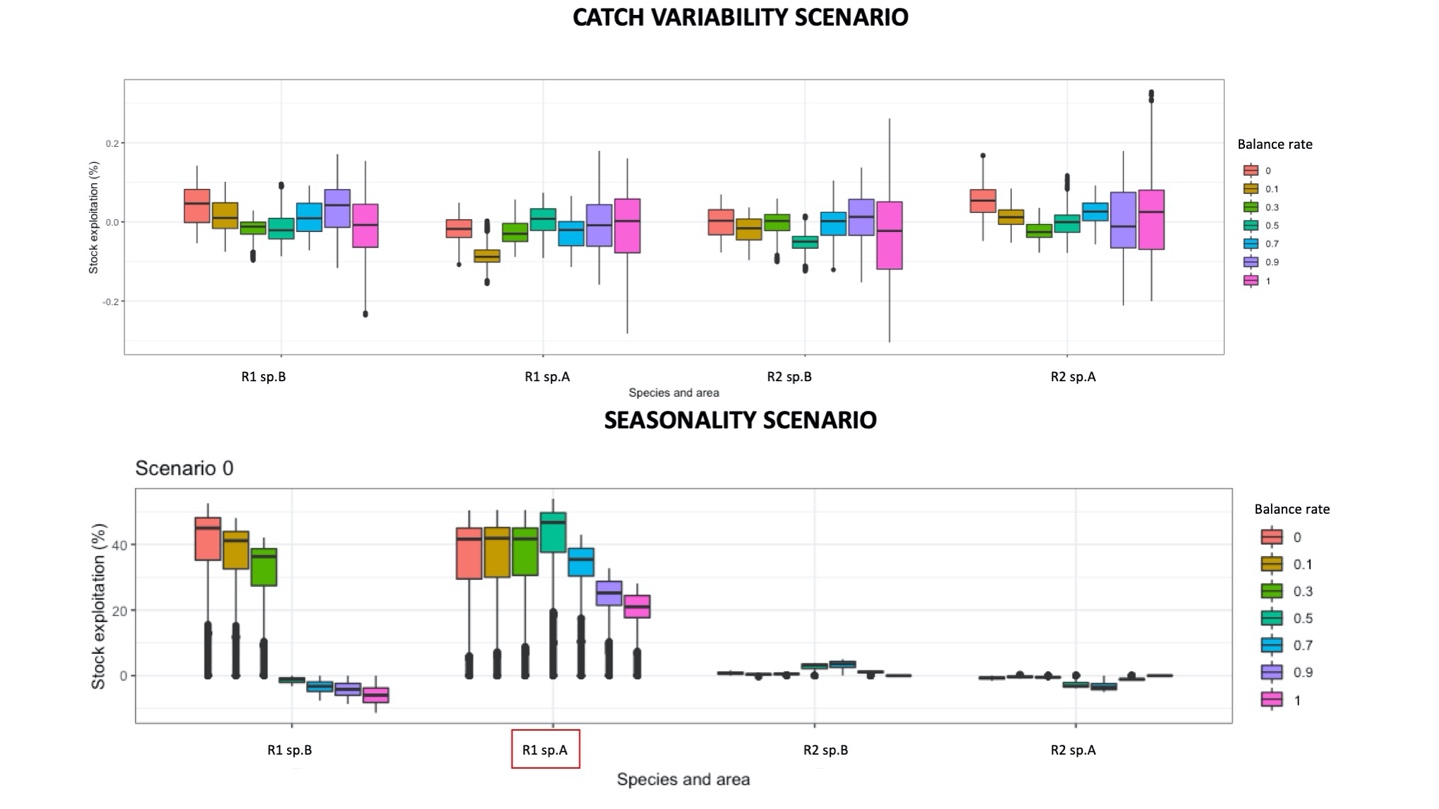


**Fig S4.9**. Average exploitation. Top panels, catch variability scenario in both regions; bottom panels, seasonality scenario (seasonality species A in region 1, indicated by red box in the Figure). Balance rate indicates the decision-making model, where demand-driven=0 (red/orange); and CPUE-driven=1 (pink).

### **Influence at the trader’s level (Fig S4.10)**

**
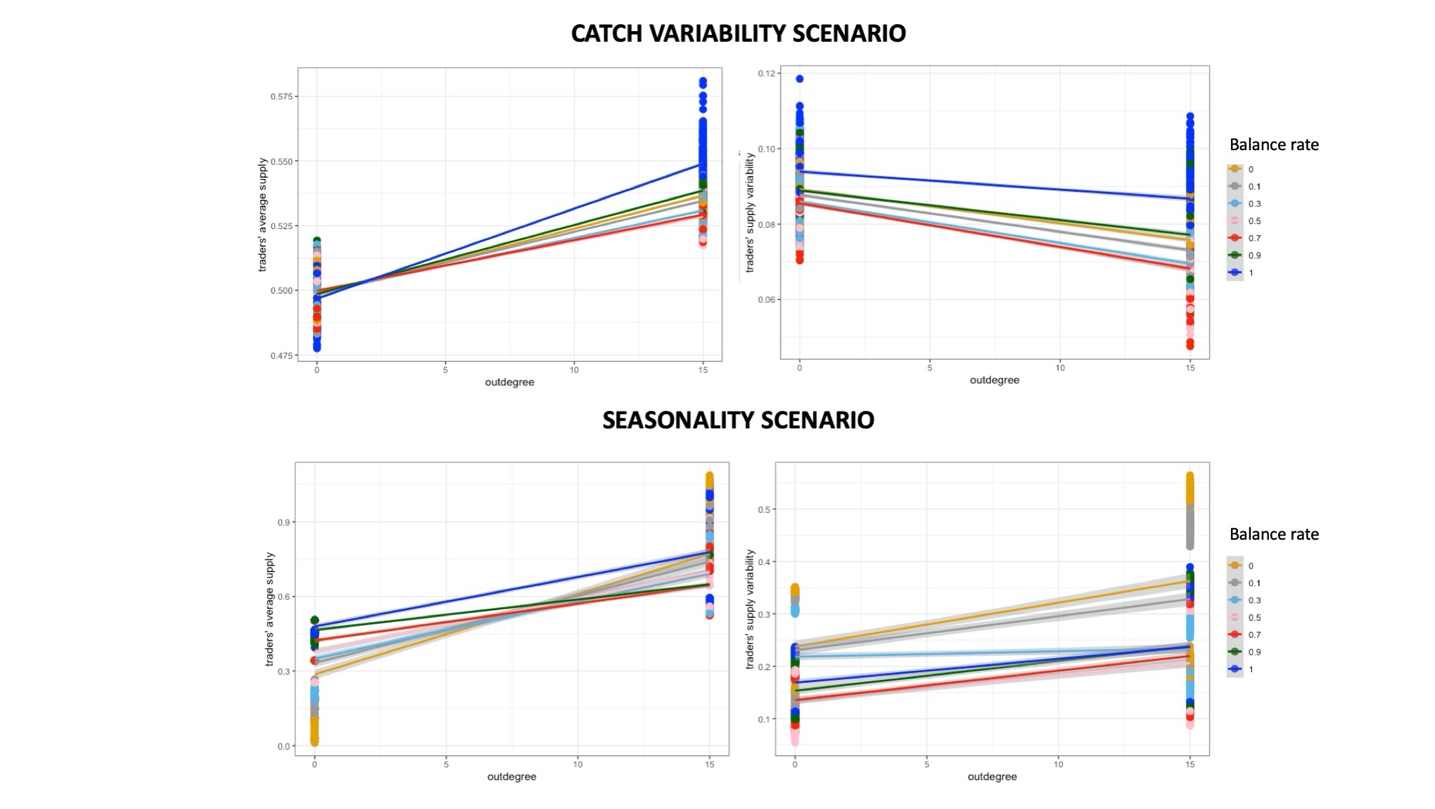
**

**Fig S4.10. Influence of the decision-making model for traders’ outcomes under the two scenarios.** Top, panel catchability scenario; bottom panel seasonality scenario (seasonality in species A region 1). Scatter plots of the average (left) and variability (right) of fish supply (considering both species types) at the trader level, as a function of the outdegree centrality of each trader, where colors represent different balance rates. Data points represent each of the 16 traders in 50 runs.

# **4. Non-aggregated time series of all network structures**

Figures below show the series for all the networks presented in the main paper, which allow verifying and understanding the model outputs. The time series are shown for 100 runs in every experiment and scenario, which are overlapped in each plot below. Showing the non-averaged time series allow to identify if different types of behaviors can emerge between runs. The Figures shown below show significant overlap between the runs and we cannot identify different behaviors. In the seasonality scenario (scenario 1), the time series look similar as in the averaged time series. In the catch variability scenario (scenario 2), we cannot identify different types of behavior but the effect of the stochasticity should be further explored.

## **Scenario 1. Seasonality (Fig S4.11 and S4.12)**

**
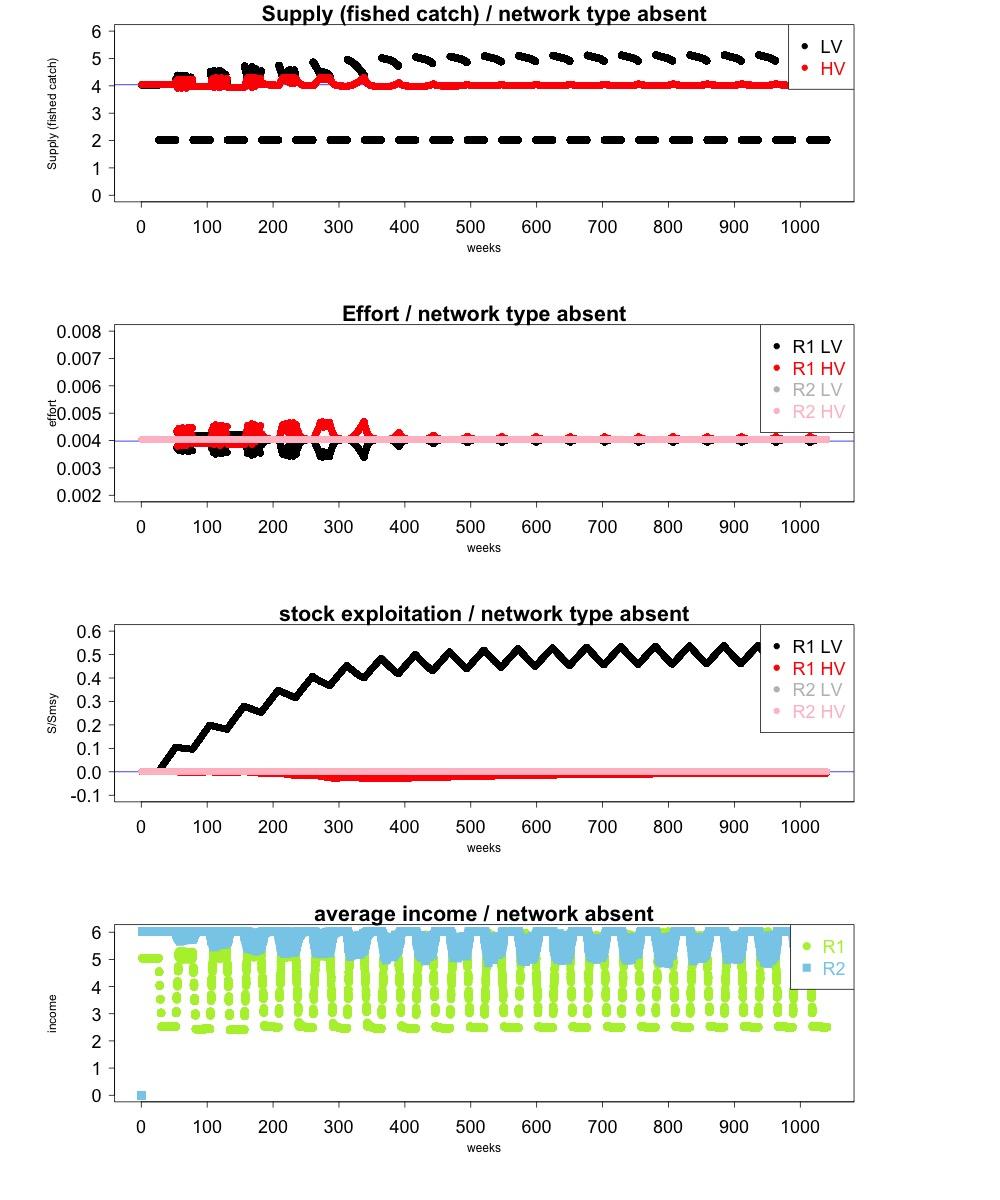
**

**Fig S4.11.** Seasonality scenario without a trade network (network type: absent). HV, species B in the manuscript; LV species A in the manuscript.


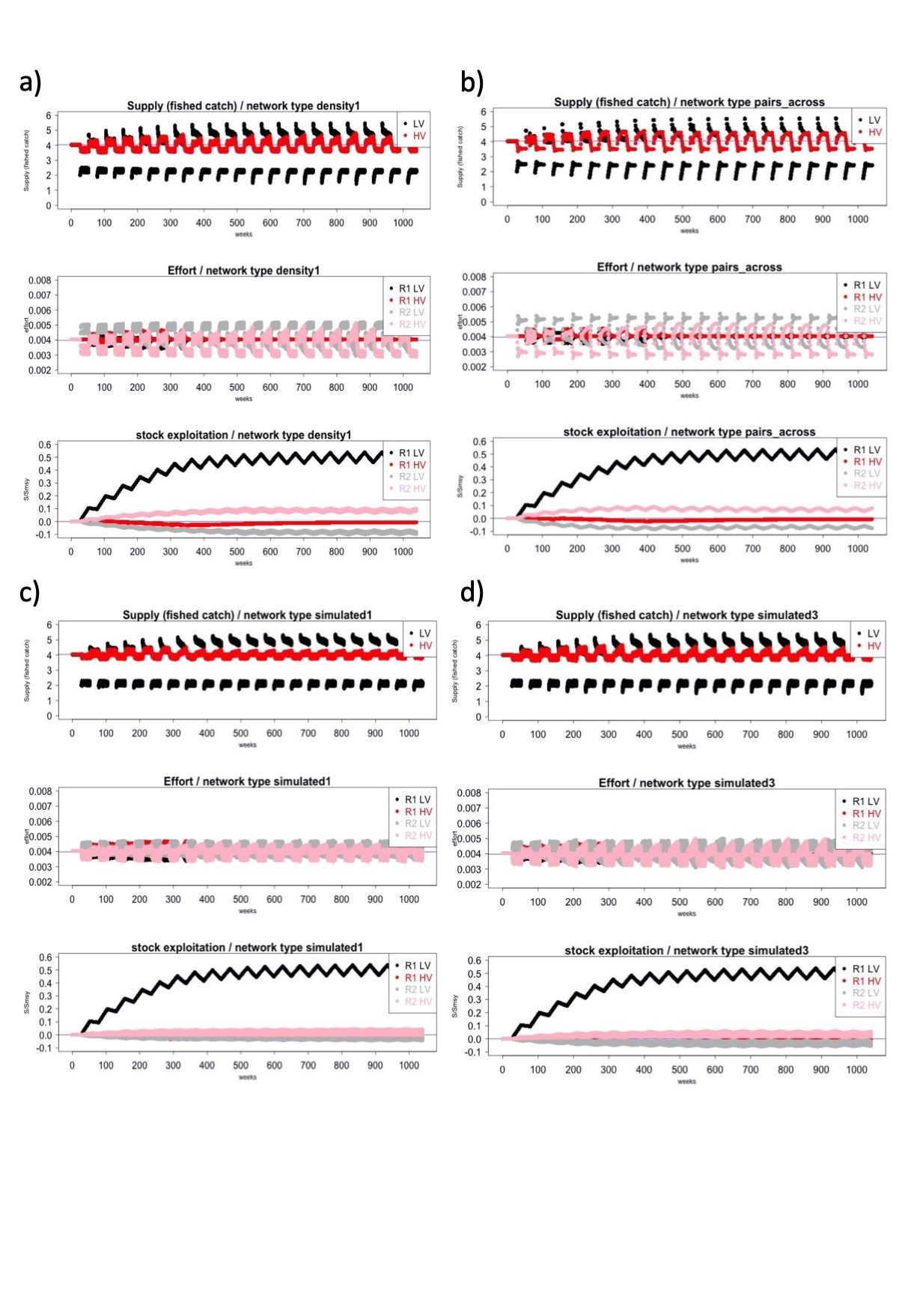


**Fig S4.12.** Seasonality scenario with different network types. HV, species B in the manuscript; LV species A in the manuscript. Network types: a) highly connected; b) pairs across; c) empirically simulated; d) more-dealers.

## **Scenario 2. Catch variability (Fig S4.13 and S4.14)**

**
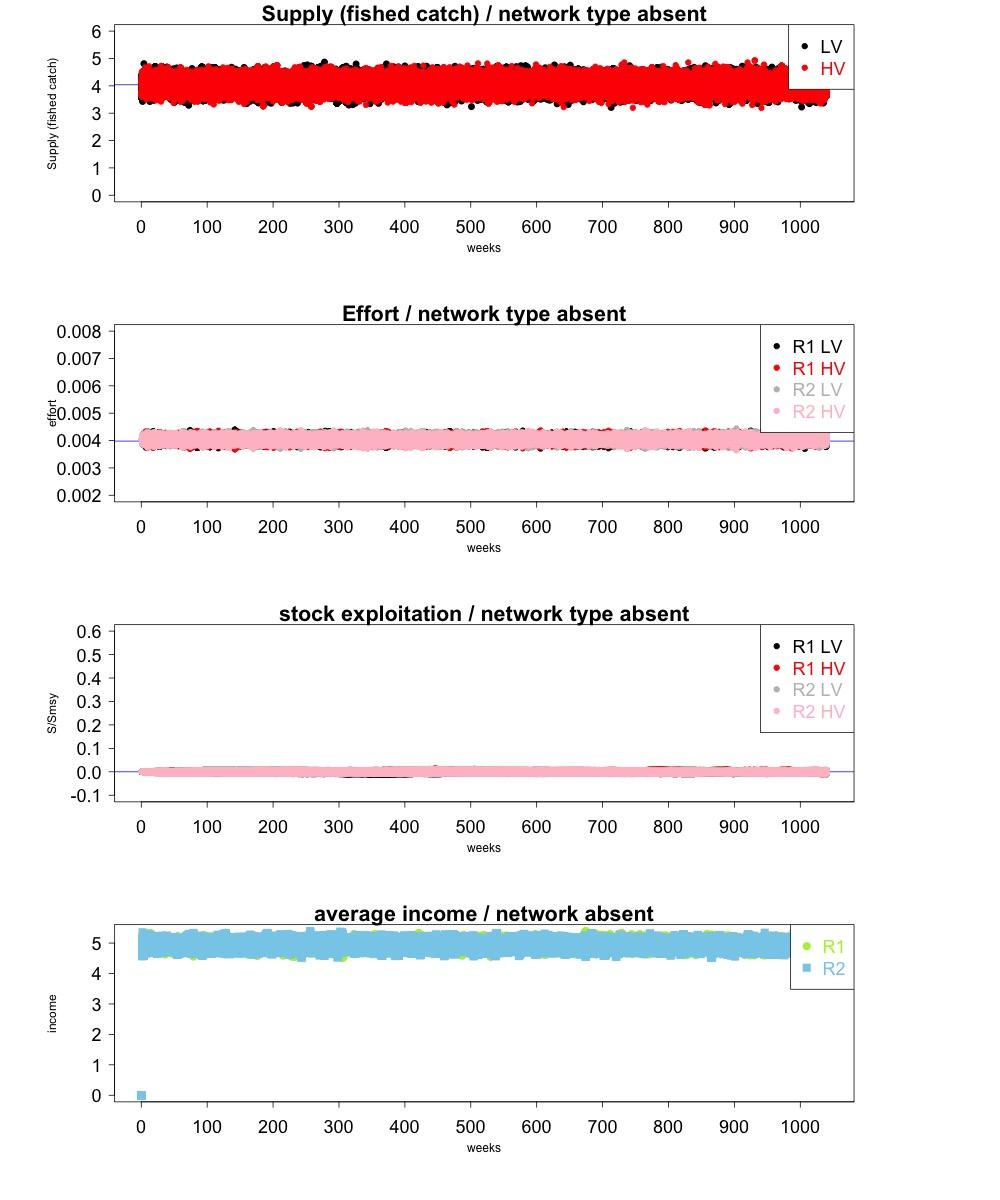
**

**Fig S4.13.** Catch variability scenario without a trade network (network type: absent). HV, species B in the manuscript; LV species A in the manuscript.

**
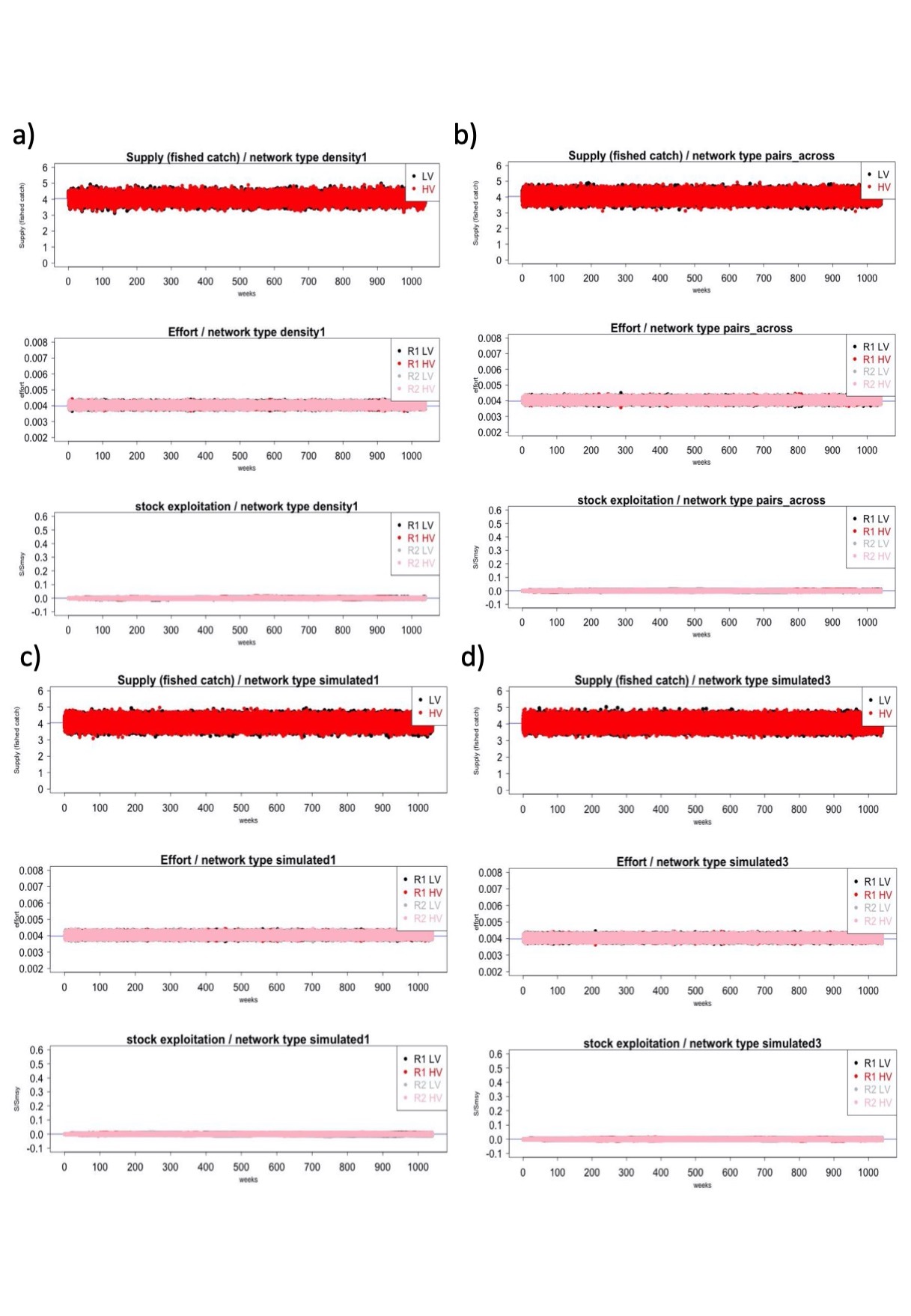
**

**Fig S4.14.** Catch variability scenario with different network types. HV, species B in the manuscript; LV species A in the manuscript. Network types: a) highly connected; b) pairs across; c) empirically simulated; d) more-dealers.
